# Prognostic Models for Global Functional Outcome and Post-Concussion Symptoms Following Mild Traumatic Brain Injury: A Collaborative European NeuroTrauma Effectiveness Research in Traumatic Brain Injury (CENTER-TBI) Study

**DOI:** 10.1089/neu.2022.0320

**Published:** 2023-08-16

**Authors:** Ana Mikolić, Ewout W. Steyerberg, Suzanne Polinder, Lindsay Wilson, Marina Zeldovich, Nicole von Steinbuechel, Virginia F.J. Newcombe, David K. Menon, Joukje van der Naalt, Hester F. Lingsma, Andrew I.R. Maas, David van Klaveren

**Affiliations:** ^1^ Department of Public Health, Erasmus MC-University Medical Center Rotterdam, Rotterdam, the Netherlands.; ^2^ Department of Biomedical Data Sciences, Leiden University Medical Center, Leiden, the Netherlands.; ^3^ Division of Psychology, University of Stirling, Stirling, United Kingdom.; ^4^ Institute of Medical Psychology and Medical Sociology, University Medical Center Göttingen, Georg-August-University, Göttingen, Germany.; ^5^ Division of Anesthesia, Department of Medicine, University of Cambridge, Cambridge, United Kingdom.; ^6^ Department of Neurology, University of Groningen, University Medical Center Groningen, the Netherlands.; ^7^ Department of Neurosurgery, Antwerp University Hospital and University of Antwerp, Edegem, Belgium.; ^8^ Predictive Analytics and Comparative Effectiveness Center, Institute for Clinical Research and Health Policy Studies/Tufts Medical Center, Boston, Massachusetts, USA.

**Keywords:** biomarkers, Glasgow Outcome Scale Extended, mild traumatic brain injury, post-concussion symptoms, predictors, prognostic model

## Abstract

After mild traumatic brain injury (mTBI), a substantial proportion of individuals do not fully recover on the Glasgow Outcome Scale Extended (GOSE) or experience persistent post-concussion symptoms (PPCS). We aimed to develop prognostic models for the GOSE and PPCS at 6 months after mTBI and to assess the prognostic value of different categories of predictors (clinical variables; questionnaires; computed tomography [CT]; blood biomarkers). From the Collaborative European NeuroTrauma Effectiveness Research in Traumatic Brain Injury (CENTER-TBI) study, we included participants aged 16 or older with Glasgow Coma Score (GCS) 13-15. We used ordinal logistic regression to model the relationship between predictors and the GOSE, and linear regression to model the relationship between predictors and the Rivermead Post-concussion Symptoms Questionnaire (RPQ) total score. First, we studied a pre-specified Core model. Next, we extended the Core model with other clinical and sociodemographic variables available at presentation (Clinical model). The Clinical model was then extended with variables assessed before discharge from hospital: early post-concussion symptoms, CT variables, biomarkers, or all three categories (extended models). In a subset of patients mostly discharged home from the emergency department, the Clinical model was extended with 2-3–week post-concussion and mental health symptoms. Predictors were selected based on Akaike's Information Criterion. Performance of ordinal models was expressed as a concordance index (C) and performance of linear models as proportion of variance explained (R2). Bootstrap validation was used to correct for optimism. We included 2376 mTBI patients with 6-month GOSE and 1605 patients with 6-month RPQ. The Core and Clinical models for GOSE showed moderate discrimination (C = 0.68 95% confidence interval 0.68 to 0.70 and C = 0.70[0.69 to 0.71], respectively) and injury severity was the strongest predictor. The extended models had better discriminative ability (C = 0.71[0.69 to 0.72] with early symptoms; 0.71[0.70 to 0.72] with CT variables or with blood biomarkers; 0.72[0.71 to 0.73] with all three categories). The performance of models for RPQ was modest (R2 = 4% Core; R2 = 9% Clinical), and extensions with early symptoms increased the R2 to 12%. The 2-3-week models had better performance for both outcomes in the subset of participants with these symptoms measured (C = 0.74 [0.71 to 0.78] vs. C = 0.63[0.61 to 0.67] for GOSE; R2 = 37% vs. 6% for RPQ). In conclusion, the models based on variables available before discharge have moderate performance for the prediction of GOSE and poor performance for the prediction of PPCS. Symptoms assessed at 2-3 weeks are required for better predictive ability of both outcomes. The performance of the proposed models should be examined in independent cohorts.

## Introduction

The majority of patients after traumatic brain injury (TBI) present with a Glasgow Coma Score (GCS) of 13 to 15 and are classified as mild.^1^ However, “mild” appears to be a misnomer since a substantial proportion of patients do not completely return to their pre-injury level of functioning and/or experience persistent post-concussion symptoms (PPCS) several months after sustaining a TBI.^2-4^ It would be beneficial to identify individuals early after injury who are at higher risk of suboptimal functional outcome or PPCS, as this would facilitate follow up for therapeutic intervention. Although high-quality evidence is still limited,^5,6^ brief early psycho-educational and cognitive-behavioral interventions have the potential to improve functional outcome and reduce the likelihood of persistent symptoms after mild TBI.^7-9^

There are currently no satisfactory models for prediction of outcomes following mild TBI.^10,11^ Our recent external validation study performed in a large European cohort of TBI patients^12^ showed that none of the models for prediction of 6-month outcome after mild TBI based on variables available at presentation had both good agreement between observed and predicted values and good ability to distinguish between individuals with favorable and unfavorable outcome. The definition of unfavorable outcome, however, differed between prognostic studies. Predicting the full Glasgow Outcome Scale Extended (GOSE)^13^ range compared with dichotomization by a cutoff would have greater statistical power and be more informative.^14^

Prognostic models that included 2-3–week symptoms had satisfactory performance at external validation.^12^ Studies in mild TBI consistently show that symptoms measured weeks after injury improve prediction and therefore should be routinely collected.^3,11,15^ Assessing 2-3–week symptoms, however, is often clinically impractical and unsuitable for acute care of mild TBI patients. There is a need for a model that can predict outcome in the acute setting, in addition to a prediction model that incorporates measures assessed later after injury.

Patient-reported symptoms measured early after a TBI (0-7 days) predict incomplete recovery and persistent symptoms after 1-3 months.^16–19^ Imaging variables have shown inconsistent associations with the functional and symptomatic outcomes, depending on other characteristics of mild TBI patients, the exact type of lesion, and definition of the outcome.^3,20^ Blood biomarkers have been associated with intracranial abnormalities on computed tomography (CT) following mild TBI^21-23^ but they have been insufficiently investigated for longer-term prognosis. If they turn out to be independent predictors of outcome, as some studies suggest,^24,25^ biomarkers would represent a readily accessible asset in the acute care after mild TBI.^26^

We aimed to develop prognostic models for GOSE and PPCS 6 months after mild TBI based on characteristics available at presentation and suitable for early detection of high-risk patients. We explored if the performance of prognostic models improved by adding different categories of predictors available before discharge from hospital: biomarkers, early post-concussion symptoms, CT characteristics, or all the aforementioned. We also explored if 2-3–week post-concussion and mental health symptoms improved the predictive performance of the models.

## Methods

### Study population

The study population consisted of participants from the prospective longitudinal observational Collaborative European NeuroTrauma Effectiveness Research in Traumatic Brain Injury (CENTER-TBI) study (registration number: NCT02210221). The data version used for this study was Core 3.0.^27^ Patients were enrolled from December 2014 to December 2017 in 63 centers across Europe and Israel. Ethical approval was obtained for each recruiting site and informed consent was obtained from all patients and/or their legal representative/next of kin: https://www.center-tbi.eu/project/ethical-approval. Inclusion criteria for the core study were a clinical diagnosis of TBI, presentation within 24 h after injury, and an indication for CT scanning according to local rules. Patients were excluded if they had severe pre-existing neurological disorder that could confound outcome assessments.^2^ In CENTER-TBI, participants were differentiated by care pathway and assigned to the emergency department (ED) stratum (discharged from an ED), admission stratum (admitted to a hospital ward), or intensive care unit (ICU) stratum (admitted to the ICU).^2^

We selected participants who were 16 years or older with a baseline GCS 13 to 15 and available outcome assessments. The majority of predictors measured at presentation were missing in <5% of included participants, but post-traumatic amnesia duration (15-17%), loss of consciousness (7-8%), education levels (9-13%), and employment (5-6%) had higher percentages of missingness (Table 1; Supplementary Table S1). CT variables were missing in 5-7%, early symptoms in 26-29%, and biomarkers in 17-20% of participants (Table 1; Supplementary Table S1). Missing predictor data were imputed to allow for fair comparison between model variants using multi-variate imputation by chained equations assuming a missingness at random mechanism.^28^ The imputation model contained all predictor and outcome variables, and predictive mean matching was used for continuous, logistic regression for binary, proportional odds logistic regression for ordinal, and polytomous regression for categorical data. For the development of models containing 2-3–week symptoms, we only selected participants for whom this assessment was obtained. By the CENTER-TBI study design, these assessments were performed in ED stratum and a subgroup of patients admitted to a hospital ward.^2^

**Table 1.** Characteristics of Mild TBI Patients With Available 6-Month Glasgow Outcome Scale Extended (GOSE; *N* = 2376) and Rivermead Post-Concussion Symptoms Questionnaire (RPQ; *n* = 1605), and 2-Week RPQ (*n* = 640; *n* = 476) in the CENTER-TBI Study

	6-month GOSE		6-month RPQ		6-Month GOSE +2-3-week RPQ		6-Month 2-3-week RPQ	
n	2376	Missing %	1605	Missing %	640	Missing %	476	Missing %
Age median [Q1, Q3]	53 [34, 68]	0	53[35, 66]	0	49[31, 62]	0	51[34.75, 63]	0
Sex male (%)	1519 (63.9)	0	1018 (63.4)	0	378 (59.1)	0	279 (58.6)	0
Pre-injury health ASA-PS (%)		0.8		0.4		0		0
No systemic disease	1292 (54.8)		909 (56.9)		383 (59.8)		383 (59.8)	
Mild	803 (34.1)		538 (33.7)		2024 (31.9)		158 (33.2)	
Severe	261 (11.1)		151 (9.4)		53 (8.3)		39 (8.2)	
Psychiatric history (%)	312 (13.3)	1	202 (12.6)	0.5	86 (13.5)	0.2	62 (13.0)	0
Cause of injury (%)		1.8		1.6		1.1		1.3
fall and other	1353 (58.0)		888 (56.2)		373 (58.9)		268 (57.0)	
Traffic	846 (36.2)		613 (38.8)		226 (35.7)		185 (39.4)	
violence	135 (5.8)		78 (4.9)		34 (5.4)		17 (3.6)	
Glasgow Coma Score (%)		0		0		0		0
13	161 (6.8)		103 (6.4)		15 (2.3)		12 (2.5)	
14	421 (17.7)		269 (16.8)		56 (8.8)		39 (8.2)	
15	1794 (75.5)		1233 (76.8)		569 (88.9)		425 (89.3)	
Total Injury Severity Score (ISS) median[Q1-Q3]	10 [5, 18]	0.9	10[5, 18]	0.7	5 [2, 9]	0.2	5[2.75, 9]	0
ISS extra-cranial	3[0,9]	0	4[0,9]	0	1[0,4]	0	1[0,4]	0
Head AIS	3[2,3]	1	3[2,3]	0.8	2[1,2]	0.3	2[1,2]	0.2
Any intracranial abnormality (%)	1028 (46.3)	6.6	718 (47.4)	5.5	113 (18.6)	5.2	98 (21.4)	3.6
NFL ≤48 h median [Q1, Q3]	13.5 [7.2, 28.5]	20.4	12.8 [7.3, 25.7]	18.2	8.4[5.2, 14.4]	16.4	8.8[5.6, 14.4]	13.7
RPQ Total Score at presentation^1^ median [Q1, Q3]	8 [2, 15]	29.4	8 [2.75, 15]	26.2	8 [2, 15.75]	10.9	8.50 [2, 16]	11.3
RPQ Total Score at 2-3 wks^2^ median [Q1, Q3]	8 [0, 20]	73.1	8[2, 21]	70.3	8[0, 20]	0	8 [2, 21]	0
RPQ Total Score at 6 months median [Q1, Q3]	6 [0, 16]	32.5	6 [0, 16]	0	4 [0, 14]	25.6	4 [0, 14]	0
GOSE at 180 days		0		0.1		0		0
1	89 (3.7)		0		1 (0.2)		0	
3	99 (4.2)		42 (2.6)		8 (1.2)		4 (0.8)	
4	74 (3.1)		53 (3.3)		1 (0.2)		1 (0.2)	
5	174 (7.3)		119 (7.4)		27 (4.2)		21 (4.4)	
6	247 (10.4)		205 (12.8)		53 (8.3)		49 (10.3)	
7	517 (21.8)		410 (25.6)		133 (20.8)		118 (24.8)	
8	1176 (49.5)		775 (48.3)		417 (65.2)		283 (59.5)	

^1^ At median 1 day post-injury [Q1:Q3: 0-1].

^2^ At median 20 days [Q1-Q3:15-28]).

TBI, traumatic brain injury; CENTER-TBI, Collaborative European NeuroTrauma Effectiveness Research in Traumatic Brain Injury; ASA- PS, American Society of Anesthesiologists Physical Status classification; AIS, Abbreviated Injury Score; NFL, neurofilament light.

### Outcomes assessed at 6 months

We analyzed associations with 6-month GOSE and PPCS. The GOSE^13^ has the following categories: 1) dead; 2) vegetative state; 3) lower severe disability; 4) upper severe disability; 5) lower moderate disability; 6) upper moderate disability; 7) lower good recovery; and 8) upper good recovery. The GOSE was collected using structured interviews and patient/caregiver questionnaires. The categories vegetative state and lower severe disability were combined in one group, as these could not be differentiated in the postal questionnaire. Overall, ∼22% had GOSE scores outside of the pre-specified 5-8-month window.^2^ We used GOSE ratings imputed to exactly 180 days based on the GOSE recorded at different time-points (from 2 weeks to 1 year) based on a multi-state model.^29^ The imputed GOSE variable was made by the CENTER-TBI statisticians and directly extracted from the CENTER-TBI dataset.^29,30^

PPCS were assessed by the Rivermead Post-Concussion Symptoms Questionnaire (RPQ).^31^ The RPQ consists of 16 common symptoms that can appear after mTBI/ concussion. Participants are asked to rate how problematic symptoms were compared with symptoms before the injury on a 5-point rating scale (0–4). A score of 0 indicates “not experienced at all”; 1 indicates “no more of a problem (than before)”; 2 indicates “a mild problem”; 3 indicates “a moderate problem”; and 4 indicates “a severe problem.” The total score is calculated as the sum of items, with a range from 0 (representing no change in symptoms since the injury) to 64 (most severe symptoms). When calculating the total score, “1” responses were rated as 0. The questionnaire was translated and linguistically validated in languages of the participating centers.^32^ When using a binary end-point, we dichotomized the RPQ Total score based on a cutoff ≥16.^33^

### Candidate predictors

#### Questionnaires

The RPQ was assessed in the hospital center (at presentation or before discharge, median 1 day [Q1-Q3:0-1]), and after 2-3-weeks in participants from ED stratum and in a subgroup of Admission stratum (median 20 days [Q1-Q3:15-28]). The Post-Traumatic Stress Disorder (PTSD) Checklist for Diagnostic and Statistical Manual of Mental Disorders, Fifth Edition (DSM)-5 (PCL-5),^34^ Generalized Anxiety Disorder 7-item scale (GAD-7),^35^ Patient Health Questionnaire (PHQ-9)^36^ were administered at 2-3 weeks, and were considered as predictors in the “2-3–week” prognostic models. The PCL-5^34^ measures symptoms of PTSD according to DSM-5 criteria. It consists of 20 items that can be answered with 0 = not at all to 5 = extremely, and it can have a score range of 0-80. The GAD-7^35^ measures severity of a general anxiety disorder. It comprises seven items that can be answered from 0 = not at all to 3 = nearly every day, and it can have a score range of 0-21. The PHQ-9^36^ measures the severity of major depressive disorder symptoms. It contains nine items using a 4-point rating scale (from 0 = not at all to 3 = nearly every day), and it can have a score range of 0-27.

#### Clinical and sociodemographic characteristics

Sociodemographic, pre-injury, and injury-related variables were prospectively collected as follows: age, GCS, total injury severity score (ISS), sex, psychiatric history, pre-injury health (American Society of Anesthesiologists Physical Status [ASA PS] Classification), prior TBI, history of migraines or headaches, education level, employment, living alone, cause of injury, alcohol intoxication, pupillary reactivity, post-traumatic amnesia, loss of consciousness, vomiting, and headache (Supplementary Table S1). GCS is the total GCS at baseline (post-stabilization value at emergency department). ISS can range from 0 to 75 (in brain-injured population from 1 to 75)^37^ and is calculated as the sum of the squares of the three body regions with the highest Abbreviated Injury Scale (AIS). If any AIS is scored 6, the ISS is automatically 75. Additionally, we calculated extra-cranial ISS (considering the AIS of the face, abdomen, chest, extremities, and external injuries, and excluding the head AIS) and head AIS (the highest AIS out of brain injury, head, neck, and cervical spine).

#### CT variables

We included the following CT characteristics, scored upon central review of the CT scans obtained at presentation: traumatic axonal injury (TAI), cisternal compression, midline shift (> 5 mm), subarachnoid hemorrhage, contusion, (non-) evacuated hematoma, and a composite variable any abnormality on CT (Supplementary Table S1).

#### Biomarkers

We included the following biomarkers sampled ≤48 h after injury: glial fibrillary acidic protein (GFAP), serum neurofilament light (NFL), neuron-specific enolase (NSE), S100 calcium-binding protein B (S100B), total-Tau (t-tau), and ubiquitin C-terminal hydrolase -L1 (UCHL1). The median sampling time was 14 h (Q1-Q3: 6-20 h). The sampling was within 24 h for the majority of patients (91%).

The sampling of blood-based biomarkers has been described in previous studies.^21^ S100B and NSE were measured with a clinical-use automated system, using an electrochemiluminescence immunoassay kit (ECLIA; Elecsys S100 and Elecsys NSE assays) run on the e602 module of Cobas 8000 modular analyzer (Roche Diagnostics, Mannheim, Germany) at the University of Pecs (Pecs, Hungary). Serum GFAP, UCHL1, NFL, and t-tau were analyzed with an ultrasensitive immunoassay using digital array technology (Single Molecule Arrays, [SiMoA]-based Human Neurology 4-Plex B assay (N4PB) run on the SR-X benchtop assay platform (Quanterix Corp., Lexington, MA) at the University of Florida (Gainesville, FL). Medians and interquartile ranges were shown for continuous variables and percentages for categorical variables (Table 1; Supplementary Table S2).

### Model development

**FIG. 1.**
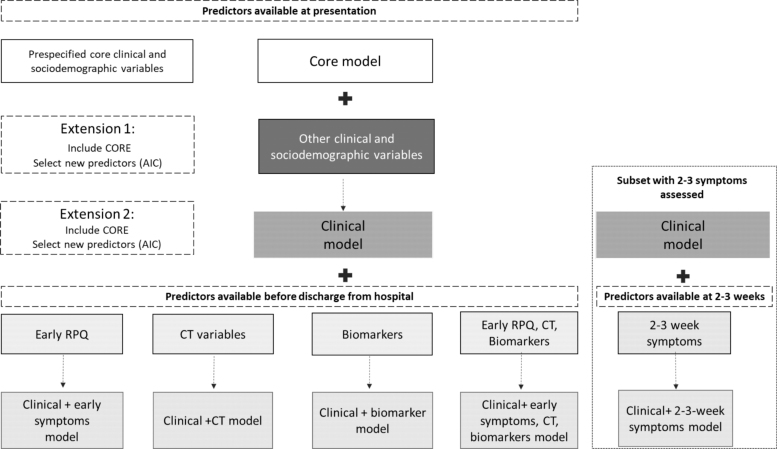
Modeling strategy.

Based on a systematic review,^38^ a recent review and validation study,^12^ subsequent studies,^17,39,40^ and clinical expertise, we selected candidate predictors and easily obtainable core variables. The selection of “core variables” was guided by the most frequent predictors from prognostic models that satisfied our methodological quality criteria.^12^ For GOSE, the core model included age, GCS, and ISS. For RPQ, the core model included sex, psychiatric history, and pre-injury health. We extended the core models with: 1) other clinical and sociodemographic variables available at presentation; 2) RPQ total score measured at presentation or before discharge; 3) CT variables; 4) blood-based biomarkers; and 5) RPQ total score, CT results, and biomarkers (Fig. 1); and 6) in the subgroup of participants in whom symptom assessments were performed at 2-3 weeks, 2-3–week post-concussion and mental health symptoms.

We used ordinal logistic regression to model the relationship between predictors and the GOSE, and linear regression to model the relationship between predictors and RPQ total score. We assessed nonlinear effects of age, ISS, and biomarkers. We assessed non-linear transformations with polynomials of log-transformed ISS and log-transformed values of biomarkers for both outcomes, and non-linear transformations with polynomials of age and of log-transformed GFAP for prediction of RPQ. When we examined extra-cranial and head injury severities separately, we assessed nonlinear transformations with polynomials for head AIS for prediction of GOSE.

**FIG. 2.**
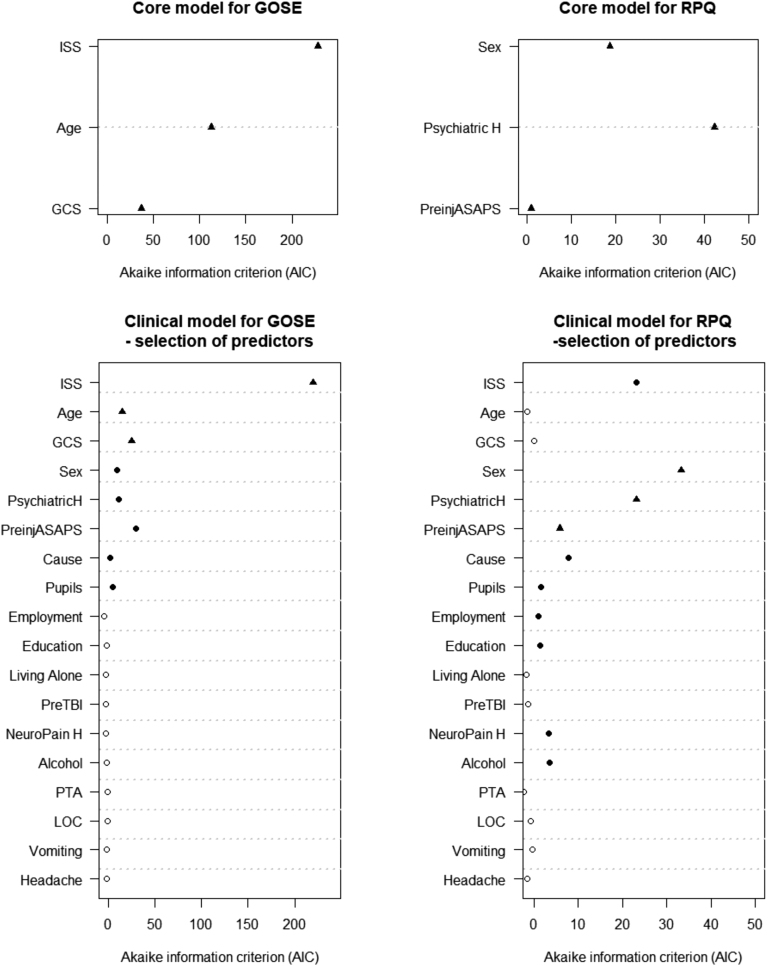
The Core and Clinical models for prediction of Glasgow Outcome Scale Extended (GOSE) and Rivermead Post-Concussion Symptom Questionnaire (RPQ). Black circles indicate selected predictors based on Akaike's Information Criterion. Black triangles indicate pre-specified core predictors. Alcohol, alcohol intoxication; ASA-PS, American Society of Anesthesiologists Physical Status; Cause, cause of injury; GCS, Glasgow Coma Scale; ISS, Injury Severity Score Total; Neuropain H, history of migraines/headaches; PreTBI, prior traumatic brain injury; PsychiatricH, psychiatric history; PTA, post-traumatic amnesia; LOC, loss of consciousness.

In the first model extension, the Core model was extended with other clinical variables (Clinical and sociodemographic characteristics). Core variables were included (“forced”) into the model and clinical predictors were selected based on Akaike's Information Criterion (AIC; Fig. 1; Fig. 2A). The AIC was used to select the best model fit with the smallest number of parameters: a higher AIC indicates better predictive ability (how much a predictor adds to the model) penalizing for the complexity of the model (as expressed by the degrees of freedom). AIC strikes a balance between identifying predictors and preventing overfitting. In the second phase, the Clinical model was extended with other categories of variables (i.e., variables 2-6 as listed above). The additional predictors were selected based on the AIC for individual factors, and core variables were always included (“forced”) into the model (Fig. 1). The AIC for candidate predictors in examined models was reported graphically.

Bootstrap validation with 500 repetitions was used to estimate a uniform shrinkage factor (corrected calibration slope) and optimism in performance. We report model equations for which the regression coefficients of the final models were multiplied by a shrinkage factor and the model intercept was re-estimated. We also report the equations of models that were refitted to a dichotomized GOSE (cutoff GOSE = 8), using the same shrinkage factor. The performance of ordinal logistic regression models was quantified with the concordance index (C), which quantifies the ability of a model to discriminate between patients with different levels of outcome. Overall performance was quantified with partial Nagelkerke R2, which represents the scaled difference in the log-likelihood of a model with and without the prognostic factor(s). The performance of the models was also reported for different cutoffs of the GOSE. The performance of linear regression models was quantified with the proportion of explained variance (R2). For comparison with other studies, we also reported C obtained in logistic regression analysis that modeled the relationship between predictors and a dichotomized RPQ Total score. Performance was calculated across imputed datasets and confidence intervals were estimated using 200 bootstrap samples.

To examine calibration of the models for predicting complete (upper-good) recovery (GOSE = 8) and significant post-concussion symptoms (RPQ ≥16) in different European regions, we performed cross-validation with a leave-one-region-out approach: The regions West, North and South-East (Supplementary Table S1) were consecutively left out for model fitting and were then used for model validation. The R-package rms^41^ (Regression Modeling Strategies) was used for all regression analyses.

## Results

### Study population

We included 2376 participants with (an imputed) GOSE at 180 days. For 1605 participants, RPQ was assessed at 6-month follow-up. The median age was 53 years and the majority of patients were male (64% and 63%) and with GCS 15 (76% and 77%; Table 1). The median ISS was 10 and almost half of the patients had intracranial abnormalities on CT (Table 1). The median RPQ Total score was 8 at baseline and 2-3-weeks, and 6 at 6 months (Table 1). About half of the participants did not completely return to their pre-injury functioning according to the GOSE. As expected, participants who had symptoms measured at 2-3 weeks (*n* = 640 for the outcome GOSE; *n* = 476 for the outcome RPQ) were less severely injured (median ISS 5 and ISS extra-cranial 1; 89% GCS 15; 19% and 21% intracranial abnormalities), younger (median age 49 and 51), and somewhat less frequently male (59%; Table 1; Supplementary Table S2).

### Prediction of 6-month GOSE

The pre-specified Core model contained age, ISS (non-linear), and GCS. It had a discriminative ability of C = 0.68 (CI 95% 0.68-0.70). All predictors contributed to the model, but ISS was by far the strongest predictor (Fig. 2A). When the model was extended with sex, pre-injury health (American Society of Anesthesiologists Physical Status), psychiatric history, cause of injury, and pupillary reactivity, the performance improved (C = 0.70, CI 95% [0.69-0.71], Nagelkerke R2 increased from 18% to 21%; Table 2; Supplementary Table S3). The strongest predictor in this Clinical model was ISS, followed by pre-injury health, GCS, and age (Fig. 2B; Supplementary Table S4). When we modeled extra-cranial injury severity (ISSe) and head injury severity (head AIS) separately, rather than the overall ISS, model performance was comparable (C = 0.70 (CI 95% [0.68-0.71]). Both predictors, but especially head AIS, were strong (Supplementary Fig. S1).

**Table 2.** Prognostic Models for 6-Month Glasgow Outcome Scale Extended (GOSE) After Mild TBI: Model Performance (*N* = 2376)

	Core model	Clinical model	Clinical +early symptoms (RPQ)	Clinical +CT	Clinical +Biomarkers	Clinical+ early symptoms, CT, biomarkers	Clinical +2-3–week symptoms [subset* n* = 640]
	Ordinal GOSE (1-8)						
Nagelkerke R2 (optimism-corrected)	0.18	0.21	0.22	0.24	0.23	0.26	0.21
C (optimism- corrected) 95% CI	0.68 [0.68-0.70]	0.70 [0.69-0.71]	0.71 [0.69-0.72]	0.71 [0.70-0.72]	0.71 [0.70 -0.72]	0.72 [0.71-0.73]	0.74 [0.71-0.78]
	C (optimism-corrected) for different cutoffs						
GOSE = 8^1^	0.69	0.70	0.72	0.71	0.71	0.73	0.75
GOSE ≥7^2^	0.73	0.74	0.75	0.75	0.76	0.76	0.76
GOSE ≥5^3^	0.79	0.80	0.80	0.80	0.82	0.81	0.69^**^

^**^ Only 10 outcome events.

^1^ Complete vs. incomplete recovery/death.

^2^ Good recovery vs. disability/death.

^3^ Moderate disability/good recovery (“favorable”) vs. severe disability/death (“unfavorable”).

TBI, traumatic brain injury; RPQ, Rivermead Post-Concussion Symptoms Questionnaire; CT, computed tomography; C, concordance index; CI, confidence Interval.

When the Clinical model was extended with either early symptoms measured at presentation or before discharge, CT variables, or biomarkers, the performance further improved to a similar degree for all models (early symptoms: C = 0.71 [0.69-0.72], Nagelkerke R2 = 22%; CT variables: C = 0.71[0.70 -0.72], Nagelkerke R2 = 24%; biomarkers: C = 0.71[0.70 -0.72], Nagelkerke R2 = 23%; Table 2; Supplementary Table S4). In all these models, ISS was the strongest predictor, and pre-injury health, age, GCS, psychiatric history, and sex also were robust predictors. In addition, early RPQ had high predictive ability and was selected for the Clinical Early symptoms model (Supplementary Fig. S2; Supplementary Table S4). Any intracranial abnormality, traumatic subarachnoid hemorrhage (tSAH), TAI, and non-evacuated hematoma were selected for the Clinical CT model (Supplementary Fig. S3; Supplementary Table S4). The final Clinical Biomarker model contained NFL, S100B, and NSE, in addition to clinical variables (Supplementary Fig. S4; Supplementary Table S4).

**FIG. 3.**
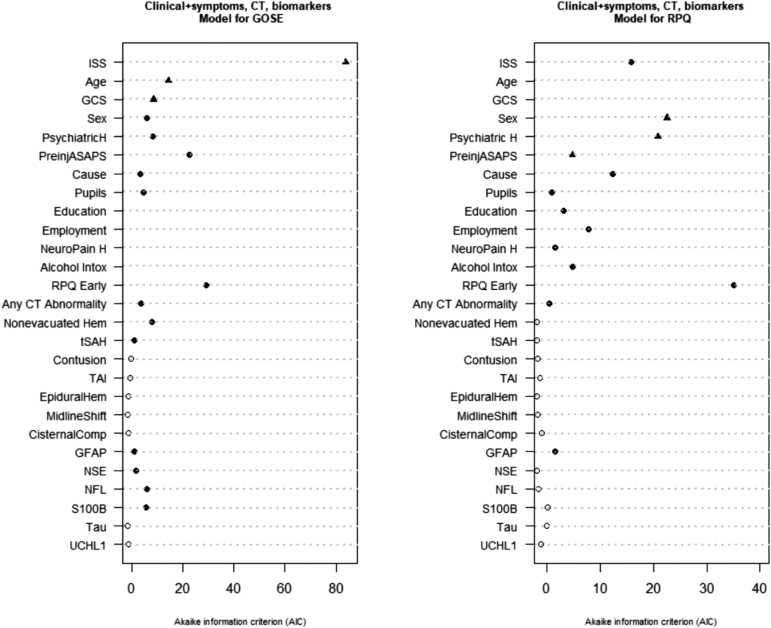
The Clinical+ symptoms, computed tomography (CT), biomarkers models for prediction of Glasgow Outcome Scale Extended (GOSE), and Rivermead Post-Concussion Symptom Questionnaire (RPQ). Black circles indicate selected predictors based on Akaike's Information Criterion. Black triangles indicate pre-specified core predictors. ASA-PS, American Society of Anesthesiologists Physical Status; Cause, cause of injury; CisternalComp, cisternal compression; GCS, Glasgow Coma Scale; Hem, hematoma; Intox, intoxication; ISS, Injury Severity Score Total; Neuropain H, history of migraines/headaches; PsychiatriH, psychiatric history; TAI, traumatic axonal injury; tSah, traumatic subarachnoid hemorrhage.

When the Clinical model was simultaneously extended with all three types of variables (early RPQ measured at presentation or before discharge, CT variables, biomarkers), the performance improved further (C = 0.72 [0.71-0.73], Nagelkerke R2 = 26%; Table 2). The final model included all variables from the Clinical model; early RPQ data; CT variables any intracranial abnormality, non-evacuated hematoma, and tSAH, and biomarkers NFL, s100B, GFAP and NSE. Consistent with other analyses, ISS, early RPQ data, and pre-injury health showed the best predictive ability (Fig. 3; Supplementary Table S4). All described models discriminated better for the outcome good recovery (GOSE ≥7; C = 0.73-0.76; Table 2) and moderate disability/good recovery versus severe disability/ death (GOSE ≥5; C = 0.79-0.82) than for upper good recovery (GOSE = 8; C = 0.69-0.73; Table 2).

**FIG. 4.**
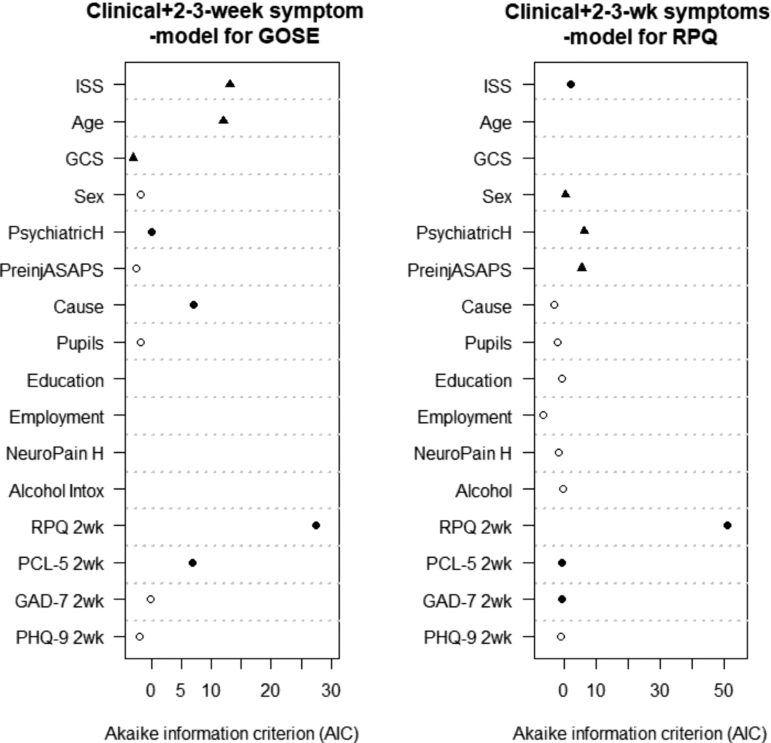
The Clinical +2-3–week symptoms models for prediction of Glasgow Outcome Scale Extended (GOSE) and Rivermead Post-Concussion Symptom Questionnaire (RPQ). Black circles indicate selected predictors based on Akaike's Information Criterion. Black triangles indicate pre-specified core predictors. ASA-PS, American Society of Anesthesiologists Physical Status; Cause, cause of injury; GAD-7, Generalized Anxiety Disorder 7-item scale (GAD-7); GCS, Glasgow Coma Scale; ISS, Injury Severity Score Total; Neuropain H, history of migraines/headaches; PCL-5, Post-Traumatic Stress Disorder (PTSD) Checklist for Diagnostic and Statistical Manual of Mental Disorders, Fifth Edition (DSM)-5; PHQ-9, Patient Health Questionnaire; PsychiatricH, psychiatric history; wk, week.

The model developed in the subset of participants with 2-3–week symptoms available had substantially better discriminative ability (C = 0.74 [0.71-0.78] compared with C = 0.63 [0.61-0.67] of the Clinical model without 2-3–week symptoms in the same subset, *n* = 640) and overall performance (Nagelkerke R2 21% vs. 7%). Apart from the core variables, this model included cause of injury, psychiatric history, post-concussion (RPQ) and post-traumatic stress disorder (PCL-5) symptoms. The strongest predictors were 2-3-week post-concussion symptoms (Fig. 4). ISS (particularly of the head) and age were also important predictors in this subset (Fig. 4; Supplementary Table S4; Supplementary Fig. S5).

The models were well-calibrated across the regions (Supplementary Fig. S6; Supplementary Fig. S7 for Clinical model). The probability of 6-month outcome can be calculated based on model equations (Box 1; Supplementary Table S5).

**Box 1.** Predicting Global Functional Outcome (GOSE) for Two Different Patients Based on the Clinical Model

**Patient 1:** Woman, 44 years, mild systemic disease (mild obesity), psychiatric history (depression), TBI caused by motor vehicle accident (MVA), GCS 14, Total ISS 9, one nonreactive pupil.
**Linear predictor (lp) =** 0.965+(-0.010^*^44)+(-0.263^*^1)+(-0.533^*^1)+(0.269^*^1)+(-0.169^*^1)+(0.099^*^log(9))+
(-0.193^*^(log(9)^2))+(-0.502^*^1)
**1/(1 + exp ^– lp^** ) = 0.2 = **20%** probability of complete return to preinjury functioning
**Patient 2:** Man, 32 years, no systemic disease, no psychiatric history, TBI caused by fall, GCS 15, Total ISS 2, reactive pupils.
**lp =** 0.965+(0.403^*^1)+(-0.010^*^32)+(0.549^*^1)+(0.099^*^log(2))+(-0.193^*^(log(2)^2))
**1/(1 + exp ^– lp^** ) = 0.83 = **83%** probability of complete recovery to preinjury functioning

GOSE, Glasgow Outcome Scale Extended; GCS; Glasgow Coma Score; ISS, Injury Severity Score.

### Prediction of 6-month RPQ

The Core model for RPQ including sex, psychiatric history and pre-injury health explained only 4% of the variance of the 6-month RPQ Total Score (Table 3; Fig. 2A). For the Clinical model, apart from the core variables, ISS, cause of injury, pupillary reactivity, alcohol intoxication, history of headaches, education, and employment were also selected. With the inclusion of new variables, the proportion of explained variance increased, but it remained modest (9%; Table 3). The strongest predictors of outcome were sex, psychiatric history, and ISS (Fig. 2B; Supplementary Table S6A, S6B). When we included ISSe and head AIS separately, the model performance was similar (R2 = 9%) and both predictors were selected; nevertheless, head AIS had a stronger predictive ability.

**Table 3.** Prognostic Models for 6-Month Rivermead Post-Concussion Symptoms Questionnaire (RPQ) Total Score After Mild TBI: Model Performance (*n* = 1605)

	Core model	Clinical model	Clinical +early symptoms (RPQ)	Clinical +CT	Clinical +Biomarker	Clinical+ early RPQ,CT, biomarkers	Clinical +2-3–week symptoms [subset* n* = 476]
R2 (optimism-corrected)	0.04	0.09	0.12	0.09	0.09	0.12	0.37
C (optimism-corrected) for cutoff ≥16^1^	0.60	0.65	0.67	0.65	0.66	0.67	0.83

^1^ More severe symptoms.

TBI, traumatic brain injury; CT, computed tomography; R2, coefficient of determination; C, concordance index.

In the extensions of the Clinical model, the proportion of explained variance increased to 12% when early symptoms (RPQ) measured at presentation/ before discharge were added, and also when all three categories were added (Table 3; Supplementary Fig. S2). Extending the models only with CT variables and biomarkers did modestly improve the model performance (R2 = 9%, Table 3). However, some predictors were selected in addition to the Clinical model: any intracranial abnormality for the Clinical CT model (Supplementary Fig. S3; Supplementary Table S5); and GFAP and Tau for the Clinical biomarker model (Supplementary Fig. S4; Supplementary Table S5). For the model extended with all three types of variables, early RPQ data, any intracranial abnormality on CT and GFAP were selected in addition to the Clinical model (Fig. 3; Supplementary Table S5). In all extended models, the strongest predictors were sex and psychiatric disorder, and other robust predictors, but to a lesser extent, were ISS, pre-injury health, and cause of injury. From additional categories, early RPQ data had a particularly strong predictive ability (Fig. 3; Supplementary Fig. S2; Supplementary Table S5).

The model developed in the subset of patients with symptoms reported at 2-3 weeks explained 37% of the variance (compared with 6% for the Clinical model in the same subset). It included, in addition to the core variables: ISS, 2-3–week post-concussion (RPQ), post-traumatic stress (PCL-5), and anxiety symptoms (GAD-7; Fig. 4; Supplementary Table S5). By far, the strongest predictor was the 2-3–week RPQ (Fig. 4). In contrast with previous analyses, male sex was associated with higher PPCS after the addition of 2-3-week symptoms (Supplementary Table S5). When we included ISSe and head AIS separately, only ISSe was selected for the Clinical model in the subset (Supplementary Fig. 5A) and only head AIS for the Clinical +2-3–week symptoms model (Supplementary Fig. 5B). The 6-month RPQ score can be estimated based on model equations (Box 2; Supplementary Table S7).

**Box 2.** Predicting Post-Concussion Symptoms (RPQ Score) for Two Different Patients Based on the Clinical Model

**Patient 1:** Woman, mild systemic disease (mild obesity), history of headaches, psychiatric history (depression), secondary education, part-time employed, TBI caused by motor vehicle accident, not intoxicated, Total ISS 9, one nonreactive pupil.
**Total RPQ score** = 7.295 (intercept) +(1.098^*^1)+(3.323^*^1)+(4.460^*^1)+(1.524^*^1)+(0.192^*^1)+(-0.709^*^log(9))+ (0.584^*^log(9)^2) +(3.910^*^1) = **24**
**Patient 2:** Man, no systemic disease, no psychiatric history, bachelor degree, full-time employed, TBI caused by fall, not intoxicated, Total ISS 2, reactive pupils.
**Total RPQ score** = 7.295+(-3.376^*^1)+(-0.709^*^log(2))+(0.584^*^(log(2 )^2) = **4**

TBI, traumatic brain injury; ISS, injury severity score.

The logistic models predicting dichotomized 6-month RPQ (cutoff ≥16) had a discriminative ability corrected for optimism between C = 0.60 and 0.67 (Table 3). Only the Clinical model with 2-3–week symptoms, developed in the subset of participants, had much better discriminative ability (C = 0.83; Table 3).

## Discussion

We developed prognostic models for global functional outcome (GOSE) and persistent post-concussion symptoms (RPQ) 6 months after mild TBI and assessed the additional value of different categories of predictors. The Clinical model for GOSE, containing age, GCS, ISS, pre-injury health, psychiatric history, cause of injury, and pupillary reactivity had moderate discriminative ability (C = 0.70), and ISS was the strongest predictor. The models extended with additional categories of predictors: early post-concussion symptoms, CT variables, blood biomarkers, and all three categories of variables had slightly better discriminative ability (C = 0.71-0.72). When the model was extended with symptoms measured at 2-3 weeks, the discriminative ability was substantially better (C = 0.74 vs. 0.63 in a subset primarily discharged home from the ED), primarily based on the strong predictive ability of post-concussion symptoms. The Clinical model for 6-month PPCS including sex, pre-injury health, psychiatric history, ISS, pupillary reactivity, alcohol intoxication, history of migraines, education, and employment explained only 9% of the outcome variance. The extension with early post-concussion symptoms increased the proportion of explained variance (to 12%), whereas the addition of CT variables and blood biomarkers did not. The model with 2-3-week symptoms had substantially better performance (R2 = 37% vs. 6% in the subset of participants with the symptoms measured).

In the CENTER-TBI study, global functional outcome could be predicted moderately well based on readily available injury-related, pre-injury, and sociodemographic characteristics, and other categories of predictors that could be collected before discharge from hospital. However, our results support the view that, based on these variables, it is easier to differentiate mild TBI patients in the lower levels of 6-month GOSE than in the highest level.^10^ Our models discriminated better for the end-point severe disability/death (GOSE <5) and disability/death (GOSE <7) than for incomplete recovery (GOSE <8), and performed better in CENTER-TBI data^12^ than the existing models developed to predict these outcomes.^42,43^ For the end-point incomplete recovery, our clinical and extended models had somewhat better performance (C = 0.70-0.73) than the UPFRONT ED model^3^ in its derivation cohort (C = 0.69) but the performance of the UPFRONT ED model could not be examined in CENTER-TBI data. In the UPFRONT study, for instance, injury severity score, one of the strongest predictors in our study, was not a candidate predictor, medical history only incorporated neurological domain, blood-based biomarkers were not assessed, and CT abnormalities were not found Predictive of the outcome. An important predictor in UPFRONT, “neck pain,” was not separately assessed in the acute stage in CENTER-TBI.

Although it has been suggested that outcome in mild TBI is primarily determined by what “the patient brings to the injury”,^10^ our result suggest that the injury severity is essential for the prediction of outcome even in mild TBI, as quantified by the high AIC for ISS in all analyses. While both head and extra-cranial injury severities were important predictors, the robustness of injury severity score in prediction of both outcomes was primarily driven by the severity of head injury. In some other mild TBI studies, however, ISS was not a strong predictor of 6-month outcome.^10^ This discrepancy could arise from differences in study populations (e.g., in the variability of ISS), other candidate predictors and outcome assessments. For instance, in mild TBI populations with overall low severity of head and non-head injuries, ISS, and intracranial lesions may not be equally informative for identifying patients at risk of worse outcomes. Further, aspects of physical and psychiatric pre-injury health also represent robust predictors of functioning after mild TBI, as shown by this and other studies.^10,44^

Early post-concussion symptoms, CT variables, and biomarkers measured before discharge from hospital further improved the performance of models for GOSE. In particular, higher early post-concussion symptoms (median 1-day post-injury) were associated with a lower likelihood of a good functional outcome. That is in line with a recent prognostic model for 1-month GOSE that incorporated acute post-concussion symptoms, such as headache, concentration difficulty, and photophobia.^17^ (Non-) evacuated hematoma, although rare in this group of patients, had high predictive value, consistent with the CRASH model for prediction of outcome in patients with GCS ≤14.^43^ Some blood biomarkers showed multivariable associations with the outcomes, but the increase in discriminative ability was not substantial. Higher levels of NFL (“chronic biomarker”) and S100B (“acute”) were associated with a lower likelihood of a good functional outcome. In previous studies, correlations were found between NFL, t-Tau, and occasionally GFAP, and return to sport, more severe symptoms, and unfavorable outcome.^45–48^ Our findings support further examination of biomarkers as predictors of outcome in mild TBI; nevertheless, they do not appear as central components of prognostic models for long-term prognosis in mild TBI. However, they can be relevant for understanding the underlying mechanisms of outcome differences. Finally, 2-3–week post-concussion symptoms were strong predictors of GOSE, which is consistent with the UPFRONT study showing improved model performance after inclusion of emotional distress and coping measured at 2 weeks (from C = 0.69 to 0.77).^3^

Similarly, as for the prediction of the incomplete recovery (GOSE = 8), the proportion of explained variance was low in the models for PPCS that did not include early and particularly 2-3–week post-concussion symptoms. Due to CENTER-TBI study design (assessment of 2-3–week symptoms only in a subgroup), we cannot draw strong conclusions, but it seems that it is not sufficient to assess symptoms only on presentation or early during hospital stay. The performance of these models (clinical and extended models) was in line with other studies.^38,49^ Even though 6-month symptoms could not be predicted well based on characteristics available before discharge from hospital, the predictors sex, psychiatric history, pre-injury health, and ISS (particularly head injury severity) showed associations with PPCS. CT positivity, and biomarkers (in particular, GFAP) were associated with PPCS, but neither CT variables nor blood biomarkers notably improved the performance of the models for PPCS. Interestingly, different biomarkers were selected in models for GOSE and PPCS. Generally, similar predictors were important for the prediction of both PPCS and GOSE; however, injury-related characteristics were more prominent predictors of the GOSE and personal/pre-injury characteristics of PPCS.

In the subset of participants primarily discharged from ED, the discriminative ability of the models containing only baseline characteristics had a lower discriminative ability. The performance of the model with 2-3–week symptoms was satisfactory and even higher than other published prognostic models for PPCS that include the symptoms^11,50^ in CENTER TBI data (C = 0.75-0.76).^12^ Therefore, in order to identify individuals with PPCS (and with incomplete recovery), the post-concussion symptoms should be assessed at follow-up. Additionally, illness perceptions^51^ and maladaptive coping^52^ have been found predictive of PPCS. A brief assessment of the most predictive symptoms could be organized in person, by telephone, or online after several weeks where feasible. Moreover, a recent model based on characteristics available at admission that showed a good discriminative ability included detailed assessments of personal factors (including personality and pre-injury status).^53^ A more comprehensive assessment at presentation or before discharge might represent a substitute or addition to a follow-up; however, it may not be practical in acute care.

### Strengths and limitations

We developed prognostic models in a large sample of contemporary patients with mild TBI. These models have shown comparable or better discriminative ability than the existing published models for mild TBI. We added different categories of predictors, which could demonstrate the incremental value of different types of variables. Moreover, the model(s) can be selected for research and clinical purposes based on the available type of data. Different categories of predictors could make the models applicable for making predictions in different clinical contexts. To prevent overfitting, we pre-specified important variables based on the literature and clinical knowledge, used favorable event per variable ratio, and used internal validation procedures. Missing values were imputed using multiple imputation. To increase power and to cover all levels of the outcome, the GOSE was analyzed as an ordinal variable. We examined models' calibration in different regions.

The CENTER-TBI participants with GCS 13-15 had a high percentage of intracranial and extra-cranial injuries. One of the inclusion criteria was an indication for CT scanning and large trauma centers were over-represented. It is possible that injury-related characteristics and CT variables would be more homogeneous in a broader patient selection who present to the ED (majority with low injury severity and without CT abnormalities), and that the models would therefore have a lower discriminative ability. That is also suggested by the poorer performance of the Clinical model in the subset of participants primarily discharged home after the ED and less severely injured. In addition, the 2-3–week symptoms were only assessed in that subset. The predictive ability of post-concussion and post-traumatic stress symptoms, important predictors of outcome, were therefore not determined in the entire spectrum of mild TBI patients. Imputed 6-month RPQ scores were not available in the database, and further work is needed to provide such scores; nonetheless, the sample size was considerable (*n* = 1605). Biomarker values and RPQ at presentation/before discharge were not available for a substantial number of participants. Further, we could not make a clear distinction between an evacuated and a non-evacuated hematoma, since the central review was blinded on the information on surgery. We considered education and employment status as candidate predictors, but socioeconomic (not available) and racial minority status (97% White) were not analyzed.

Core variables were defined based on other published models for mild TBI and researchers' judgement. Therefore, the selection of these predictors may have been somewhat arbitrary. Nevertheless, the predictors specified as core generally showed considerable predictors' effects (as shown by AIC), and therefore we did not think that this choice substantially impacted the selection of predictors and model performance. Importantly, whereas in CENTER-TBI the GOSE rating included assessments of the consequence of all injuries, including extra-cranial, in some contexts (e.g., trials in the United States), the GOSE typically includes an assessment of the consequences of TBI only.^54^ That can impact the importance of some predictors, such as injury severity score and biomarkers. Biomarkers GFAP, NFL, UCHL1, and Tau were analyzed on a research platform not commercially available, which impedes the validation and usage of the models in which they were included. Finally, these models have not yet been validated in an independent cohort.

### Conclusion

We presented prognostic models for the prediction of GOSE and PPCS 6 months after mild TBI. The models for GOSE based on predictors available before discharge from the hospital have moderate performance and ISS is the strongest predictor. In a subset of mild TBI patients who present to the ED and have less intracranial and extracranial injuries, these models have lower discriminative ability. The models for PPCS without post-injury symptoms perform poorly. CT variables, biomarkers (NFL and S100B), and questionnaires assessing symptoms improve predictions of GOSE, and questionnaires assessing symptoms improve predictions of PPCS. For both outcomes, the models with symptoms assessed at 2-3 weeks have substantially better performance, which should encourage scheduling follow-up appointments. The examination of the performance of the proposed models in independent cohorts is warranted.

## Transparency, Rigor, and Reproducibility Summary

The CENTER-TBI study was registered with ClinicalTrials.gov (NCT02210221). The analytic plan for the current study was approved by the CENTER-TBI Management Committee and registered after data collection but before data analysis at: https://www.center-tbi.eu/data/approved-proposals. The total number of CENTER-TBI Core study participants was 4509. Out of 2864 participants considered eligible for this study (age 16+ years and baseline Glasgow Coma Score 13-15), *n* = 2376 had registered 6-month Glasgow Outcome Scale Extended (GOSE) and *n* = 1605 completed 6-month Rivermead Post-concussion Symptoms Questionnaire (RPQ). By the CENTER-TBI study design, 2-3–week symptoms were assessed in a subset of participants. The total number of predictor parameters considered for the development of prognostic models was 43, which was considered adequate for the effective sample size (*n* = 2376 for ordinal GOSE, subset *n* = 640; *n* = 1605 for RPQ total score, subset *n* = 476). Trained reviewers of CT scans were blinded to clinical information except for sex, age, and care path stratum. Qualified laboratory technicians who analyzed biomarker assays were blinded to clinical information. Outcome assessments were administered by local staff and responses were subsequently entered by them on an electronic case report form. Local investigators were not blinded to acute clinical information; however, the GOSE and RPQ were both scored centrally. Outcome instruments and validated translations used in the study are available at: https://www.center-tbi.eu/project/validated-translations-outcome-instruments. The handling of missing data and analytical decisions are described in the text. The cross-validation of the models was performed in different European regions with a leave-one-out approach, and external validation was not performed. Model equations necessary for external validation are reported in the supplementary material. De-identified CENTER-TBI data, including the subset used for this study, will be available to researchers who provide a methodologically sound study proposal for review (submitted at: https://www.center-tbi.eu/data) and approval by the Management Committee. A data use agreement is required, which must comply with current regulatory requirements. This paper will be published under a Creative Commons Open Access license, and upon publication will be freely available at https://www.liebertpub.com/loi/neu.

## Ethical Approval

The CENTER-TBI study (EC grant 602150) has been conducted in accordance with all relevant laws of the European Union if directly applicable or of direct effect and all relevant laws of the country where the recruiting sites were located, including but not limited to, the relevant privacy and data protection laws and regulations (the ‘‘Privacy Law’’), the relevant laws and regulations on the use of human materials, and all relevant guidance relating to clinical studies from time to time in force including, but not limited to, the ICH Harmonised Tripartite Guideline for Good Clinical Practice (CPMP/ICH/135/95) (‘‘ICH GCP’’) and the World Medical Association Declaration of Helsinki entitled ‘‘Ethical Principles for Medical Research Involving Human Subjects’’. Informed consent by the patients and/or the legal representative/next of kin was obtained, according to the local legislations, for all patients recruited in the core data set of CENTER-TBI and documented in the electronic case report form (e-CRF). Ethical approval was obtained for each recruiting site. The list of sites, ethical committees, approval numbers, and approval dates can be found on the Web site https://www.centertbi.eu/project/ethical-approval

## The CENTER-TBI Participants and Investigators

Cecilia Åkerlund, Department of Physiology and Pharmacology, Section of Perioperative Medicine and Intensive Care, Karolinska Institutet, Stockholm, Sweden; Krisztina Amrein, János Szentágothai Research Centre, University of Pécs, Pécs, Hungary; Nada Andelic, Division of Surgery and Clinical Neuroscience, Department of Physical Medicine and Rehabilitation, Oslo University Hospital and University of Oslo, Oslo, Norway; Lasse Andreassen, Department of Neurosurgery, University Hospital Northern Norway, Tromso, Norway; Audny Anke, Department of Physical Medicine and Rehabilitation, University Hospital Northern Norway, Tromso, Norway; Anna Antoni, Trauma Surgery, Medical University Vienna, Vienna, Austria; Gérard Audibert, Department of Anesthesiology and Intensive Care, University Hospital Nancy, Nancy, France; Philippe Azouvi, Raymond Poincare Hospital, Assistance Publique—Hopitaux de Paris, Paris, France; Maria Luisa Azzolini, Department of Anesthesiology and Intensive Care, S Raffaele University Hospital, Milan, Italy; Ronald Bartels, Department of Neurosurgery, Radboud University Medical Center, Nijmegen, the Netherlands; Pál Barzó, Department of Neurosurgery, University of Szeged, Szeged, Hungary; Romuald Beauvais, International Projects Management, ARTTIC, Munchen, Germany; Ronny Beer, Department of Neurology, Neurological Intensive Care Unit, Medical University of Innsbruck, Innsbruck, Austria; Bo-Michael Bellander, Department of Neurosurgery and Anesthesia and Intensive Care Medicine, Karolinska University Hospital, Stockholm, Sweden; Antonio Belli, National Institute for Health and Care Research Surgical Reconstruction and Microbiology Research Centre, Birmingham, U.K.; Habib Benali, Anesthesie-Réanimation, Assistance Publique—Hopitaux de Paris, Paris, France; Maurizio Berardino, Department of Anesthesia and ICU, AOU Città della Salute e della Scienza di Torino-Orthopedic and Trauma Center, Torino, Italy; Luigi Beretta, Department of Anesthesiology and Intensive Care, S Raffaele University Hospital, Milan, Italy; Morten Blaabjerg, Department of Neurology, Odense University Hospital, Odense, Denmark; Peter Bragge, BehaviourWorks Australia, Monash Sustainability Institute, Monash University, Victoria, Australia; Alexandra Brazinova, Department of Public Health, Faculty of Health Sciences and Social Work, Trnava University, Trnava, Slovakia; Vibeke Brinck, Quesgen Systems Inc., Burlingame, CA, USA; Joanne Brooker, Australian and New Zealand Intensive Care Research Centre, Department of Epidemiology and Preventive Medicine, School of Public Health and Preventive Medicine, Monash University, Melbourne, Australia; Camilla Brorsson, Department of Surgery and Perioperative Science, Umeå University, Umeå, Sweden; Andras Buki, Department of Neurosurgery, Medical School, University of Pécs, Hungary and Neurotrauma Research Group, János Szentágothai Research Centre, University of Pécs, Hungary; Monika Bullinger, Department of Medical Psychology, Universitätsklinikum Hamburg-Eppendorf, Hamburg, Germany; Manuel Cabeleira, Brain Physics Lab, Division of Neurosurgery, Department of Clinical Neurosciences, University of Cambridge, Addenbrooke's Hospital, Cambridge, U.K.; Alessio Caccioppola, Neuro ICU, Fondazione IRCCS Cà Granda Ospedale Maggiore Policlinico, Milan, Italy; Emiliana Calappi, Neuro ICU, Fondazione IRCCS Cà Granda Ospedale Maggiore Policlinico, Milan, Italy; Maria Rosa Calvi, Department of Anesthesiology and Intensive Care, S Raffaele University Hospital, Milan, Italy; Peter Cameron, Australian and New Zealand Intensive Care Research Centre, Monash University, Department of Epidemiology and Preventive Medicine, Melbourne, Victoria, Australia; Guillermo Carbayo Lozano, Department of Neurosurgery, Hospital of Cruces, Bilbao, Spain; Marco Carbonara, Neuro ICU, Fondazione IRCCS Cà Granda Ospedale Maggiore Policlinico, Milan, Italy; Simona Cavallo, Department of Anesthesia and ICU, AOU Città della Salute e della Scienza di Torino-Orthopedic and Trauma Center, Torino, Italy; Giorgio Chevallard, NeuroIntensive Care, Niguarda Hospital, Milan, Italy; Arturo Chieregato, NeuroIntensive Care, Niguarda Hospital, Milan, Italy; Giuseppe Citerio, School of Medicine and Surgery, Università Milano Bicocca, Milano, Italy, NeuroIntensive Care, ASST di Monza, Monza, Italy; Hans Clusmann, Department of Neurosurgery, Medical Faculty RWTH Aachen University, Aachen, Germany; Mark Coburn, Department of Anesthesiology and Intensive Care Medicine, University Hospital Bonn, Bonn, Germany; Jonathan Coles, Department of Anesthesia and Neurointensive Care, Cambridge University Hospital NHS Foundation Trust, Cambridge, U.K.; Jamie D. Cooper, School of Public Health and PM, Monash University and the Alfred Hospital, Melbourne, Victoria, Australia; Marta Correia, Radiology/MRI department, MRC Cognition and Brain Sciences Unit, Cambridge, U.K.; Amra Čović, Institute of Medical Psychology and Medical Sociology, Universitätsmedizin Göttingen, Göttingen, Germany; Nicola Curry, Oxford University Hospitals NHS Trust, Oxford, U.K.; Endre Czeiter, Department of Neurosurgery, Medical School, University of Pécs, Hungary and Neurotrauma Research Group, János Szentágothai Research Centre, University of Pécs, Hungary; Marek Czosnyka, Brain Physics Lab, Division of Neurosurgery, Department of Clinical Neurosciences, University of Cambridge, Addenbrooke's Hospital, Cambridge, U.K.; Claire Dahyot Fizelier, Intensive Care Unit, CHU Poitiers, Potiers, France; Paul Dark, University of Manchester NIHR Biomedical Research Centre, Critical Care Directorate, Salford Royal Hospital NHS Foundation Trust, Salford, U.K.; Helen Dawes, Movement Science Group, Faculty of Health and Life Sciences, Oxford Brookes University, Oxford, U.K.; Véronique De Keyser, Department of Neurosurgery, Antwerp University Hospital and University of Antwerp, Edegem, Belgium; Vincent Degos, Anesthesie-Réanimation, Assistance Publique—Hopitaux de Paris, Paris, France; Francesco Della Corte, Department of Anesthesia and Intensive Care, Maggiore Della Carità Hospital, Novara, Italy; Hugo den Boogert, Department of Neurosurgery, Radboud University Medical Center, Nijmegen, the Netherlands; Bart Depreitere, Department of Neurosurgery, University Hospitals Leuven, Leuven, Belgium; Đula Đilvesi, Department of Neurosurgery, Clinical Centre of Vojvodina, Faculty of Medicine, University of Novi Sad, Novi Sad, Serbia; Abhishek Dixit, Division of Anaesthesia, University of Cambridge, Addenbrooke's Hospital, Cambridge, U.K.; Emma Donoghue, Australian and New Zealand Intensive Care Research Centre, Department of Epidemiology and Preventive Medicine, School of Public Health and Preventive Medicine, Monash University, Melbourne, Australia; Jens Dreier, Center for Stroke Research Berlin, Charité—Universitätsmedizin Berlin, corporate member of Freie Universität Berlin, Humboldt-Universität zu Berlin, and Berlin Institute of Health, Berlin, Germany; Guy Loup Dulière, Intensive Care Unit, CHR Citadelle, Liège, Belgium; Ari Ercole, Division of Anaesthesia, University of Cambridge, Addenbrooke's Hospital, Cambridge, U.K.; Patrick Esser, Movement Science Group, Faculty of Health and Life Sciences, Oxford Brookes University, Oxford, U.K.; Erzsébet Ezer, Department of Anaesthesiology and Intensive Therapy, University of Pécs, Pécs, Hungary; Martin Fabricius, Departments of Neurology, Clinical Neurophysiology and Neuroanesthesiology, Region Hovedstaden Rigshospitalet, Copenhagen, Denmark; Valery L. Feigin, National Institute for Stroke and Applied Neurosciences, Faculty of Health and Environmental Studies, Auckland University of Technology, Auckland, New Zealand; Kelly Foks, Department of Neurology, Erasmus MC, Rotterdam, the Netherlands; Shirin Frisvold, Department of Anesthesiology and Intensive Care, University Hospital Northern Norway, Tromso, Norway; Alex Furmanov, Department of Neurosurgery, Hadassah-hebrew University Medical center, Jerusalem, Israel; Pablo Gagliardo, Fundación Instituto Valenciano de Neurorrehabilitación (FIVAN), Valencia, Spain; Damien Galanaud, Anesthesie-Réanimation, Assistance Publique—Hopitaux de Paris, Paris, France; Dashiell Gantner, Australian and New Zealand Intensive Care Research Centre, Monash University, Department of Epidemiology and Preventive Medicine, Melbourne, Victoria, Australia; Guoyi Gao, Department of Neurosurgery, Shanghai Renji Hospital, Shanghai Jiaotong University/School of Medicine, Shanghai, China; Pradeep George, Karolinska Institutet, INCF International Neuroinformatics Coordinating Facility, Stockholm, Sweden; Alexandre Ghuysen, Emergency Department, CHU, Liège, Belgium; Lelde Giga, Neurosurgery Clinic, Pauls Stradins Clinical University Hospital, Riga, Latvia; Ben Glocker, Department of Computing, Imperial College London, London, U.K., Jagoš Golubovic, Department of Neurosurgery, Clinical Centre of Vojvodina, Faculty of Medicine, University of Novi Sad, Novi Sad, Serbia; Pedro A. Gomez, Department of Neurosurgery, Hospital Universitario 12 de Octubre, Madrid, Spain; Johannes Gratz, Department of Anesthesia, Critical Care and Pain Medicine, Medical University of Vienna, Austria; Benjamin Gravesteijn, Department of Public Health, Erasmus Medical Center-University Medical Center, Rotterdam, the Netherlands; Francesca Grossi, Department of Anesthesia and Intensive Care, Maggiore Della Carità Hospital, Novara, Italy; Russell L. Gruen, College of Health and Medicine, Australian National University, Canberra, Australia; Deepak Gupta, Department of Neurosurgery, Neurosciences Centre and JPN Apex Trauma Centre, All India Institute of Medical Sciences, New Delhi, India; Juanita A. Haagsma, Department of Public Health, Erasmus Medical Center-University Medical Center, Rotterdam, the Netherlands; Iain Haitsma, Department of Neurosurgery, Erasmus MC, Rotterdam, the Netherlands; Raimund Helbok, Department of Neurology, Neurological Intensive Care Unit, Medical University of Innsbruck, Innsbruck, Austria; Eirik Helseth, Department of Neurosurgery, Oslo University Hospital, Oslo, Norway; Lindsay Horton, Division of Psychology, University of Stirling, Stirling, U.K.; Jilske Huijben, Department of Public Health, Erasmus Medical Center-University Medical Center, Rotterdam, the Netherlands; Peter J. Hutchinson, Division of Neurosurgery, Department of Clinical Neurosciences, Addenbrooke's Hospital and University of Cambridge, Cambridge, U.K.; Bram Jacobs, Department of Neurology, University of Groningen, University Medical Center Groningen, Groningen, the Netherlands; Stefan Jankowski, Neurointensive Care, Sheffield Teaching Hospitals NHS Foundation Trust, Sheffield, U.K.; Mike Jarrett, Quesgen Systems Inc., Burlingame, California, USA; Ji yao Jiang, Karolinska Institutet, INCF International Neuroinformatics Coordinating Facility, Stockholm, Sweden; Faye Johnson, Salford Royal Hospital NHS Foundation Trust Acute Research Delivery Team, Salford, U.K.; Kelly Jones, National Institute for Stroke and Applied Neurosciences, Faculty of Health and Environmental Studies, Auckland University of Technology, Auckland, New Zealand; Mladen Karan, Department of Neurosurgery, Clinical Centre of Vojvodina, Faculty of Medicine, University of Novi Sad, Novi Sad, Serbia; Angelos G. Kolias, Division of Neurosurgery, Department of Clinical Neurosciences, Addenbrooke's Hospital University of Cambridge, Cambridge, U.K.; Erwin Kompanje, Department of Intensive Care and Department of Ethics and Philosophy of Medicine, Erasmus Medical Center, Rotterdam, the Netherlands; Daniel Kondziella, Departments of Neurology, Clinical Neurophysiology and Neuroanesthesiology, Region Hovedstaden Rigshospitalet, Copenhagen, Denmark; Evgenios Kornaropoulos, Division of Anaesthesia, University of Cambridge, Addenbrooke's Hospital, Cambridge, U.K.; Lars Owe Koskinen, Department of Clinical Neuroscience, Neurosurgery, Umeå University, Umeå, Sweden; Noémi Kovács, Hungarian Brain Research Program-Grant No. KTIA_13_NAP-A-II/8, University of Pécs, Pécs, Hungary; Ana Kowark, Department of Anaesthesiology, University Hospital of Aachen, Aachen, Germany; Alfonso Lagares, Department of Neurosurgery, Hospital Universitario 12 de Octubre, Madrid, Spain; Linda Lanyon, Karolinska Institutet, INCF International Neuroinformatics Coordinating Facility, Stockholm, Sweden; Steven Laureys, Cyclotron Research Center, University of Liège, Liège, Belgium; Fiona Lecky, Centre for Urgent and Emergency Care Research (CURE), Health Services Research Section, School of Health and Related Research (ScHARR), University of Sheffield, Sheffield, U.K. and Emergency Department, Salford Royal Hospital, Salford U.K.; Didier Ledoux, Cyclotron Research Center, University of Liège, Liège, Belgium; Rolf Lefering, Institute of Research in Operative Medicine (IFOM), Witten/Herdecke University, Cologne, Germany; Valerie Legrand, VP Global Project Management CNS, ICON, Paris, France; Aurelie Lejeune, Department of Anesthesiology-Intensive Care, Lille University Hospital, Lille, France; Leon Levi, Department of Neurosurgery, Rambam Medical Center, Haifa, Israel; Roger Lightfoot, Department of Anesthesiology and Intensive Care, University Hospitals Southhampton NHS Trust, Southhampton, U.K.; Hester Lingsma, Department of Public Health, Erasmus Medical Center-University Medical Center, Rotterdam, the Netherlands; Andrew I.R. Maas, Department of Neurosurgery, Antwerp University Hospital and University of Antwerp, Edegem, Belgium; Ana M. Castaño León, Department of Neurosurgery, Hospital Universitario 12 de Octubre, Madrid, Spain; Marc Maegele, Cologne-Merheim Medical Center (CMMC), Department of Traumatology, Orthopedic Surgery and Sportmedicine, Witten/Herdecke University, Cologne, Germany; Marek Majdan, Department of Public Health, Faculty of Health Sciences and Social Work, Trnava University, Trnava, Slovakia; Alex Manara, Intensive Care Unit, Southmead Hospital, Bristol, Bristol, U.K.; Geoffrey Manley, Department of Neurological Surgery, University of California, San Francisco, California, USA; Costanza Martino, Department of Anesthesia and Intensive Care, M. Bufalini Hospital, Cesena, Italy; Hugues Maréchal, Intensive Care Unit, CHR Citadelle, Liège, Belgium; Julia Mattern, Department of Neurosurgery, University Hospital Heidelberg, Heidelberg, Germany; Catherine McMahon, Department of Neurosurgery, the Walton Centre NHS Foundation Trust, Liverpool, U.K.; Béla Melegh, Department of Medical Genetics, University of Pécs, Pécs, Hungary; David Menon, Division of Anaesthesia, University of Cambridge, Addenbrooke's Hospital, Cambridge, U.K.; Tomas Menovsky, Department of Neurosurgery, Antwerp University Hospital and University of Antwerp, Edegem, Belgium; Ana Mikolic, Department of Public Health, Erasmus Medical Center-University Medical Center, Rotterdam, the Netherlands; Benoit Misset, Cyclotron Research Center, University of Liège, Liège, Belgium; Visakh Muraleedharan, Karolinska Institutet, INCF International Neuroinformatics Coordinating Facility, Stockholm, Sweden; Lynnette Murray, Australian and New Zealand Intensive Care Research Centre, Monash University, Department of Epidemiology and Preventive Medicine, Melbourne, Victoria, Australia; Ancuta Negru, Department of Neurosurgery, Emergency County Hospital Timisoara, Timisoara, Romania; David Nelson, Department of Physiology and Pharmacology, Section of Perioperative Medicine and Intensive Care, Karolinska Institutet, Stockholm, Sweden; Virginia Newcombe, Division of Anaesthesia, University of Cambridge, Addenbrooke's Hospital, Cambridge, U.K.; Daan Nieboer, Department of Public Health, Erasmus Medical Center-University Medical Center, Rotterdam, the Netherlands; József Nyirádi, János Szentágothai Research Centre, University of Pécs, Pécs, Hungary; Otesile Olubukola, Centre for Urgent and Emergency Care Research (CURE), Health Services Research Section, School of Health and Related Research (ScHARR), University of Sheffield, Sheffield, U.K.; Matej Oresic, School of Medical Sciences, Örebro University, Örebro, Sweden; Fabrizio Ortolano, Neuro ICU, Fondazione IRCCS Cà Granda Ospedale Maggiore Policlinico, Milan, Italy; Aarno Palotie, Institute for Molecular Medicine Finland, University of Helsinki, Helsinki, Finland, Analytic and Translational Genetics Unit, Department of Medicine, Psychiatric and Neurodevelopmental Genetics Unit, Department of Psychiatry, Department of Neurology, Massachusetts General Hospital, Boston, MA, USA and Program in Medical and Population Genetics, the Stanley Center for Psychiatric Research, the Broad Institute of MIT and Harvard, Cambridge, MA, USA; Paul M. Parizel, Department of Radiology, University of Antwerp, Edegem, Belgium; Jean François Payen, Department of Anesthesiology and Intensive Care, University Hospital of Grenoble, Grenoble, France; Natascha Perera, International Projects Management, ARTTIC, Munchen, Germany; Vincent Perlbarg, Anesthesie-Réanimation, Assistance Publique—Hopitaux de Paris, Paris, France; Paolo Persona, Department of Anesthesia and Intensive Care, Azienda Ospedaliera Università di Padova, Padova, Italy; Wilco Peul, Department of Neurosurgery, Leiden University Medical Center, Leiden, the Netherlands and Department of Neurosurgery, Medical Center Haaglanden, the Hague, the Netherlands; Anna Piippo-Karjalainen, Department of Neurosurgery, Helsinki University Central Hospital, Helsinki, Finland; Matti Pirinen, Institute for Molecular Medicine Finland, University of Helsinki, Helsinki, Finland; Dana Pisica, Department of Public Health, Erasmus Medical Center-University Medical Center, Rotterdam, the Netherlands; Horia Ples, Department of Neurosurgery, Emergency County Hospital Timisoara, Timisoara, Romania; Suzanne Polinder, Department of Public Health, Erasmus Medical Center-University Medical Center, Rotterdam, the Netherlands; Inigo Pomposo, Department of Neurosurgery, Hospital of Cruces, Bilbao, Spain; Jussi P. Posti, Division of Clinical Neurosciences, Department of Neurosurgery and Turku Brain Injury Centre, Turku University Hospital and University of Turku, Turku, Finland; Louis Puybasset, Department of Anesthesiology and Critical Care, Pitié-Salpêtrière Teaching Hospital, Assistance Publique, Hôpitaux de Paris and University Pierre et Marie Curie, Paris, France; Andreea Radoi, Neurotraumatology and Neurosurgery Research Unit (UNINN), Vall d'Hebron Research Institute, Barcelona, Spain; Arminas Ragauskas, Department of Neurosurgery, Kaunas University of Technology and Vilnius University, Vilnius, Lithuania; Rahul Raj, Department of Neurosurgery, Helsinki University Central Hospital, Helsinki, Finland; Malinka Rambadagalla, Department of Neurosurgery, Rezekne Hospital, Latvia, Lithuania; Isabel Retel Helmrich, Department of Public Health, Erasmus Medical Center-University Medical Center, Rotterdam, the Netherlands; Jonathan Rhodes, Department of Anaesthesia, Critical Care and Pain Medicine NHS Lothian and University of Edinburg, Edinburgh, U.K.; Sylvia Richardson, Director, MRC Biostatistics Unit, Cambridge Institute of Public Health, Cambridge, U.K.; Sophie Richter, Division of Anaesthesia, University of Cambridge, Addenbrooke's Hospital, Cambridge, U.K.; Samuli Ripatti, Institute for Molecular Medicine Finland, University of Helsinki, Helsinki, Finland; Saulius Rocka, Department of Neurosurgery, Kaunas University of Technology and Vilnius University, Vilnius, Lithuania; Cecilie Roe, Department of Physical Medicine and Rehabilitation, Oslo University Hospital/University of Oslo, Oslo, Norway; Olav Roise, Division of Orthopedics, Oslo University Hospital, Oslo, Norway and Institute of Clinical Medicine, Faculty of Medicine, University of Oslo, Oslo, Norway; Jonathan Rosand, Broad Institute, Cambridge MA Harvard Medical School, Boston, MA, USA and Massachusetts General Hospital, Boston MA, USA; Jeffrey V. Rosenfeld, National Trauma Research Institute, the Alfred Hospital, Monash University, Melbourne, Victoria, Australia; Christina Rosenlund, Department of Neurosurgery, Odense University Hospital, Odense, Denmark; Guy Rosenthal, Department of Neurosurgery, Hadassah-Hebrew University Medical center, Jerusalem, Israel; Rolf Rossaint, Department of Anaesthesiology, University Hospital of Aachen, Aachen, Germany; Sandra Rossi, Department of Anesthesia and Intensive Care, Azienda Ospedaliera Università di Padova, Padova, Italy; Daniel Rueckert, Department of Computing, Imperial College London, London, U.K.; Martin Rusnák, International Neurotrauma Research Organisation, Vienna, Austria; Juan Sahuquillo, Neurotraumatology and Neurosurgery Research Unit (UNINN), Vall d'Hebron Research Institute, Barcelona, Spain; Oliver Sakowitz, Department of Neurosurgery, University Hospital Heidelberg, Heidelberg, Germany and Klinik für Neurochirurgie, Klinikum Ludwigsburg, Ludwigsburg, Germany; Renan Sanchez Porras, Klinik für Neurochirurgie, Klinikum Ludwigsburg, Ludwigsburg, Germany; Janos Sandor, Division of Biostatistics and Epidemiology, Department of Preventive Medicine, University of Debrecen, Debrecen, Hungary; Nadine Schäfer, Institute of Research in Operative Medicine (IFOM), Witten/Herdecke University, Cologne, Germany; Silke Schmidt, Department Health and Prevention, University Greifswald, Greifswald, Germany; Herbert Schoechl, Department of Anaesthesiology and Intensive Care, AUVA Trauma Hospital, Salzburg, Austria; Guus Schoonman, Department of Neurology, Elisabeth-TweeSteden Ziekenhuis, Tilburg, the Netherlands; Rico Frederik Schou, Department of Neuroanesthesia and Neurointensive Care, Odense University Hospital, Odense, Denmark; Elisabeth Schwendenwein, Trauma Surgery, Medical University Vienna, Vienna, Austria; Charlie Sewalt, Department of Public Health, Erasmus Medical Center-University Medical Center, Rotterdam, the Netherlands; Toril Skandsen, Department of Neuromedicine and Movement Science, Norwegian University of Science and Technology, NTNU, Trondheim, Norway and Department of Physical Medicine and Rehabilitation, St. Olavs Hospital, Trondheim University Hospital, Trondheim, Norway; Peter Smielewski, Brain Physics Lab, Division of Neurosurgery, Department of Clinical Neurosciences, University of Cambridge, Addenbrooke's Hospital, Cambridge, U.K.; Abayomi Sorinola, Department of Neurosurgery, University of Pécs, Pécs, Hungary; Emmanuel Stamatakis, Division of Anaesthesia, University of Cambridge, Addenbrooke's Hospital, Cambridge, U.K.; Simon Stanworth, Oxford University Hospitals NHS Trust, Oxford, U.K.; Robert Stevens, Division of Neuroscience Critical Care, John Hopkins University School of Medicine, Baltimore, USA; William Stewart, Department of Neuropathology, Queen Elizabeth University Hospital and University of Glasgow, Glasgow, U.K.; Ewout W. Steyerberg, Department of Public Health, Erasmus Medical Center-University Medical Center, Rotterdam, the Netherlands and Department of Biomedical Data Sciences, Leiden University Medical Center, Leiden, the Netherlands; Nino Stocchetti, Department of Pathophysiology and Transplantation, Milan University and Neuroscience ICU, Fondazione IRCCS Cà Granda Ospedale Maggiore Policlinico, Milano, Italy; Nina Sundström, Department of Radiation Sciences, Biomedical Engineering, Umeå University, Umeå, Sweden; Riikka Takala, Perioperative Services, Intensive Care Medicine and Pain Management, Turku University Hospital and University of Turku, Turku, Finland; Viktória Tamás, Department of Neurosurgery, University of Pécs, Pécs, Hungary; Tomas Tamosuitis, Department of Neurosurgery, Kaunas University of Health Sciences, Kaunas, Lithuania; Mark Steven Taylor, Department of Public Health, Faculty of Health Sciences and Social Work, Trnava University, Trnava, Slovakia; Braden Te Ao, National Institute for Stroke and Applied Neurosciences, Faculty of Health and Environmental Studies, Auckland University of Technology, Auckland, New Zealand; Olli Tenovuo, Division of Clinical Neurosciences, Department of Neurosurgery and Turku Brain Injury Centre, Turku University Hospital and University of Turku, Turku, Finland; Alice Theadom, National Institute for Stroke and Applied Neurosciences, Faculty of Health and Environmental Studies, Auckland University of Technology, Auckland, New Zealand; Matt Thomas, Intensive Care Unit, Southmead Hospital, Bristol, Bristol, U.K.; Dick Tibboel, Intensive Care and Department of Pediatric Surgery, Erasmus Medical Center, Sophia Children's Hospital, Rotterdam, the Netherlands; Marjolein Timmers, Department of Intensive Care and Department of Ethics and Philosophy of Medicine, Erasmus Medical Center, Rotterdam, the Netherlands; Christos Tolias, Department of Neurosurgery, Kings college London, London, U.K.; Tony Trapani, Australian and New Zealand Intensive Care Research Centre, Monash University, Department of Epidemiology and Preventive Medicine, Melbourne, Victoria, Australia; Cristina Maria Tudora, Department of Neurosurgery, Emergency County Hospital Timisoara, Timisoara, Romania; Andreas Unterberg, Department of Neurosurgery, University Hospital Heidelberg, Heidelberg, Germany; Peter Vajkoczy, Neurologie, Neurochirurgie und Psychiatrie, Charité—Universitätsmedizin Berlin, Berlin, Germany; Shirley Vallance, Australian and New Zealand Intensive Care Research Centre, Monash University, Department of Epidemiology and Preventive Medicine, Melbourne, Victoria, Australia; Egils Valeinis, Neurosurgery Clinic, Pauls Stradins Clinical University Hospital, Riga, Latvia; Zoltán Vámos, Department of Anaesthesiology and Intensive Therapy, University of Pécs, Pécs, Hungary; Mathieu van der Jagt, Department of Intensive Care Adults, Erasmus MC—University Medical Center Rotterdam, Rotterdam, the Netherlands; Gregory Van der Steen, Department of Neurosurgery, Antwerp University Hospital and University of Antwerp, Edegem, Belgium; Joukje van der Naalt, Department of Neurology, University of Groningen, University Medical Center Groningen, Groningen, the Netherlands; Jeroen T.J.M. van Dijck, Department of Neurosurgery, Leiden University Medical Center, Leiden, the Netherlands and Department of Neurosurgery, Medical Center Haaglanden, the Hague, the Netherlands; Thomas A. van Essen, Department of Neurosurgery, Leiden University Medical Center, Leiden, the Netherlands and Department of Neurosurgery, Medical Center Haaglanden, the Hague, the Netherlands; Wim Van Hecke, icoMetrix NV, Leuven, Belgium; Caroline van Heugten, Movement Science Group, Faculty of Health and Life Sciences, Oxford Brookes University, Oxford, U.K.; Dominique Van Praag, Psychology Department, Antwerp University Hospital, Edegem, Belgium; Ernest van Veen, Department of Public Health, Erasmus Medical Center-University Medical Center, Rotterdam, the Netherlands; Thijs Vande Vyvere, icoMetrix NV, Leuven, Belgium; Roel P. J. van Wijk, Department of Neurosurgery, Leiden University Medical Center, Leiden, the Netherlands and Department of Neurosurgery, Medical Center Haaglanden, the Hague, the Netherlands; Alessia Vargiolu, NeuroIntensive Care, ASST di Monza, Monza, Italy; Emmanuel Vega, Department of Anesthesiology-Intensive Care, Lille University Hospital, Lille, France; Kimberley Velt, Department of Public Health, Erasmus Medical Center-University Medical Center, Rotterdam, the Netherlands; Jan Verheyden, icoMetrix NV, Leuven, Belgium; Paul M. Vespa, Director of Neurocritical Care, University of California, Los Angeles, USA; Anne Vik, Department of Neuromedicine and Movement Science, Norwegian University of Science and Technology, NTNU, Trondheim, Norway and Department of Neurosurgery, St. Olavs Hospital, Trondheim University Hospital, Trondheim, Norway; Rimantas Vilcinis, Department of Neurosurgery, Kaunas University of Health Sciences, Kaunas, Lithuania; Victor Volovici, Department of Neurosurgery, Erasmus MC, Rotterdam, the Netherlands; Nicole von Steinbüchel, Institute of Medical Psychology and Medical Sociology, Universitätsmedizin Göttingen, Göttingen, Germany; Daphne Voormolen, Department of Public Health, Erasmus Medical Center-University Medical Center, Rotterdam, the Netherlands; Petar Vulekovic, Department of Neurosurgery, Clinical Centre of Vojvodina, Faculty of Medicine, University of Novi Sad, Novi Sad, Serbia; Kevin K.W. Wang, Department of Emergency Medicine, University of Florida, Gainesville, Florida, USA; Eveline Wiegers, Department of Public Health, Erasmus Medical Center-University Medical Center, Rotterdam, the Netherlands; Guy Williams, Division of Anaesthesia, University of Cambridge, Addenbrooke's Hospital, Cambridge, U.K.; Lindsay Wilson, Division of Psychology, University of Stirling, Stirling, U.K.; Stefan Winzeck, Division of Anaesthesia, University of Cambridge, Addenbrooke's Hospital, Cambridge, U.K.; Stefan Wolf, Department of Neurosurgery, Charité—Universitätsmedizin Berlin, corporate member of Freie Universität Berlin, Humboldt-Universität zu Berlin, and Berlin Institute of Health, Berlin, Germany; Zhihui Yang, Broad Institute, Cambridge MA Harvard Medical School, Boston, MA, USA and Massachusetts General Hospital, Boston, MA, USA; Peter Ylén, VTT Technical Research Centre, Tampere, Finland; Alexander Younsi, Department of Neurosurgery, University Hospital Heidelberg, Heidelberg, Germany; Frederick A. Zeiler, Division of Anaesthesia, University of Cambridge, Addenbrooke's Hospital, Cambridge, U.K. and Section of Neurosurgery, Department of Surgery, Rady Faculty of Health Sciences, University of Manitoba, Winnipeg, Manitoba, Canada; Veronika Zelinkova, Department of Public Health, Faculty of Health Sciences and Social Work, Trnava University, Trnava, Slovakia; Agate Ziverte, Neurosurgery Clinic, Pauls Stradins Clinical University Hospital, Riga, Latvia; Tommaso Zoerle, Neuro ICU, Fondazione IRCCS Cà Granda Ospedale Maggiore Policlinico, Milan, Italy.

## Supplementary Material

Supplemental data

Supplemental data

Supplemental data

Supplemental data

Supplemental data

Supplemental data

Supplemental data

Supplemental data

Supplemental data

Supplemental data

Supplemental data

Supplemental data

Supplemental data

Supplemental data
