# Supplementary material for: Prognostic Models for Global Functional Outcome and Post-Concussion Symptoms Following Mild Traumatic Brain Injury: A Collaborative European NeuroTrauma Effectiveness Research in Traumatic Brain Injury (CENTER-TBI) Study
Source: J Neurotrauma. 2023 Aug 16;40(15-16):1651–70. doi: 10.1089/neu.2022.0320 (PMC10458380; doi:10.1089/neu.2022.0320)
Supplement: Supplemental data [file Supp_TableS1.docx]

**Supplementary Table 1. Categories and definitions of clinical and sociodemographic predictors, any intracranial abnormality and regions**

| **Candidate predictors** | **Unit/ categories/range** | **Definition/ notes** |
| --- | --- | --- |
| Age | Years |  |
| Sex | Male/ female |  |
| Glasgow Coma Score | 13, 14, 15 | Baseline (post-stabilization value at emergency department), if missing imputed using IMPACT methodology. |
| Injury severity score | 1-75 | Sum of the squares of 3 body regions (out of 6 main body regions) with the highest Abbreviated Injury Score. Any AIS of 6 automatically gives ISS of 75. |
| Injury severity score Extra cranial | 1-75 | Sum of the squares of 3 body regions (out of 5 main body regions, excluding the head) with the highest Abbreviated Injury Score. Any AIS of 6 automatically gives ISS of 75. |
| Abbreviated injury score Head | 1-6 | The highest scoring in the area brain, head, neck, and cervical spine. |
| Psychiatric history | Yes/no | Yes=Depression, anxiety, sleeping disorder, schizophrenia, substance abuse, other |
| Physical health ASAPS | No systemic disease/ mild systemic disease/ severe or life-threatening | ASAPS classification system used in anesthesia. |
| Prior TBI | Yes/ no | Self- report of previous TBIs/ concussions. |
| History of migraines/headaches | Yes/ no | Self- report of headaches, migraines and family history of migraine. |
| Education level | Primary school or none/secondary/ in program/ college or university degree | In program= currently in diploma or degree-oriented program, post-high training. |
| Employment | Full time/ part-time, special employment, sick leave/ student/ retired/ unemployed | Full time= 35 hours of more. Part-time= 20-34 hours per week, less than 20 hours, special employment, currently on sick leave.  Unemployed= unemployed, looking for work, homemaker. |
| Living alone | Yes/ no |  |
| Cause of injury | Traffic/ violence/ fall and other | Traffic= motor vehicle accident or other traffic.  Violence= assault, mass violence, suicide attempt.  Fall and other= incidental fall, other nonintentional injury, work or sport. |
| Alcohol intoxication | Yes/no | Yes= yes or suspected |
| Pupillary reactivity | Reactive pupils/ one or two nonreactive pupils |  |
| Posttraumatic amnesia | no/ <2h/ >2 h |  |
| Loss of consciousness | Yes/no | Yes= yes or suspected |
| Vomiting | Yes/no | At the arrival to ER, once or more. |
| Headache | Yes/no | Headache at the ER. |
| Any abnormality on CT | Yes/no | Yes=mass lesion, extra-axial and epidural hematoma, acute and subacute chronic subdural hematoma, subdural collection mixed density, contusion, traumatic axonal injury (TAI), traumatic subarachnoid (tSAH) and intraventricular hemorrhage, midline shift or cisternal compression |
| **Clusters** |  |  |
| Regions | West/ North/ South-east | West: Austria, The Netherlands, Belgium, Germany, Great Britain, France (AT, NL, BE, DE, GB, FR). North: Finland, Norway, Sweden, Denmark, Lithuania, Latvia (FI, NO, SE, DK, LV, LT). South-East: Italy, Spain, Israel, Hungary, Romania, Serbia (IT, ES, IL, HU, RO, SR). |
