# Supplementary material for: Prognostic Models for Global Functional Outcome and Post-Concussion Symptoms Following Mild Traumatic Brain Injury: A Collaborative European NeuroTrauma Effectiveness Research in Traumatic Brain Injury (CENTER-TBI) Study
Source: J Neurotrauma. 2023 Aug 16;40(15-16):1651–70. doi: 10.1089/neu.2022.0320 (PMC10458380; doi:10.1089/neu.2022.0320)
Supplement: Supplemental data [file Supp_TableS2.docx]

**Supplementary Table 2. Characteristics of mild traumatic brain injury (TBI) patients with available 6-month Glasgow Outcome Scale Extended (GOSE) (N=2376) and Rivermead Post-Concussion Symptoms Questionnaire (RPQ) (N=1605)**

|  | **6-month GOSE** | **Missing %** | **6-month RPQ** | **Missing %** |
| --- | --- | --- | --- | --- |
| n | 2376 |  | 1605 |  |
| Education levels (%) |  | 12.7 |  | 9.3 |
| none or primary | 319 (15.4) |  | 206 (14.2) |  |
| secondary | 683 (32.9) |  | 463 (31.8) |  |
| program | 484 (23.3) |  | 343 (23.6) |  |
| college/university | 589 (28.4) |  | 443 (30.4) |  |
| Employment (%) |  | 6 |  | 5.4 |
| full-time | 893 (40.0) |  | 642 (42.3) |  |
| part-time, special | 255 (11.4) |  | 200 (13.2) |  |
| retired | 666 (29.8) |  | 418 (27.5) |  |
| student | 211 (9.4) |  | 141 (9.3) |  |
| unemployed | 209 (9.4) |  | 117 (7.7) |  |
| Living Alone (%) | 529 (22.3) | 0 | 344 (21.4) | 0 |
| Previous TBI (%) | 253 (11.2) | 4.6 | 178 (11.5) | 3.9 |
| Pupils (%) | 62 (2.7) | 4.5 | 29 (1.9) | 4.8 |
| History of migraines and headaches (%) | 86 (3.7) | 1 | 66 (4.1) | 0.6 |
| Alcohol Intoxication (%) | 491 (21.6) | 4.5 | 306 (19.8) | 3.9 |
| Posttraumatic amnesia (%) |  | 16.8 |  | 15.1 |
| no | 1139 (57.6) |  | 746 (54.8) |  |
| <2h | 535 (27.1) |  | 407 (29.9) |  |
| >2h | 303 (15.3) |  | 209 (15.3) |  |
| Loss of consciousness (%) | 1304 (59.4) | 7.7 | 907 (60.7) | 6.9 |
| Vomiting (%) | 398 (17.3) | 3.2 | 255 (16.3) | 2.7 |
| Headache = 1 (%) | 575 (26.3) | 8 | 377 (25.4) | 7.4 |
| Traumatic Axonal Injury (%) | 97 (4.4) | 6.6 | 72 (4.7) | 5.5 |
| Epidural Hematoma (%) | 173 (7.8) | 6.7 | 124 (8.2) | 5.5 |
| Midline Shift (%) | 95 (4.3) | 6.6 | 48 (3.2) | 5.5 |
| (Non-)evacuated Hematoma (%) | 180 (8.1) | 6.9 | 99 (6.6) | 5.9 |
| Traumatic Subarachnoid Hemorrhage (%) | 707 (31.9) | 6.8 | 500 (33.0) | 5.5 |
| Cisternal Compression (%) | 112 (5.0) | 6.6 | 58 (3.8) | 5.5 |
| Contusion (%) | 462 (20.9) | 6.9 | 301 (19.9) | 5.9 |
| GFAP (median [Q1, Q3]) | 1.60 [0.32, 6.29] | 20.4 | 1.71 [0.35, 6.31] | 18.2 |
| NFL (median [Q1, Q3]) | 13.46 [7.15, 28.48] | 20.4 | 12.80 [7.28, 25.68] | 18.2 |
| NSE (median [Q1, Q3]) | 14.50 [11.49, 20.08] | 19.3 | 14.49 [11.49, 20.08] | 16.9 |
| S100B (median [Q1, Q3]) | 0.10 [0.06, 0.20] | 19.2 | 0.10 [0.06, 0.19] | 16.9 |
| UCHL1 (median [Q1, Q3]) | 59.03 [27.98, 147.55] | 20.7 | 58.61 [28.22, 144.41] | 18.6 |
| Tau (median [Q1, Q3]) | 1.84 [1.00, 3.98] | 20.3 | 1.81 [1.02, 3.82] | 18.1 |
| PCL5 Total Score (median [Q1, Q3]) | 9.00 [3.00, 19.00] | 73.8 | 9.00 [3.00, 19.25] | 70.8 |
| PHQ9 Total Score (median [Q1, Q3]) | 5.00 [1.00, 10.00] | 73.7 | 5.00 [2.00, 10.00] | 70.9 |
| GAD7 Total Score (median [Q1, Q3]) | 2.00 [0.00, 6.00] | 73.7 | 2.00 [0.00, 6.00] | 71 |

**Legend:** GAD-7= Generalized Anxiety Disorder 7-item scale; PHQ-9**=** Patient Health Questionnaire; PCL-5**=**Post-Traumatic Stress Disorder (PTSD) Checklist for DSM-5.
