# Supplementary material for: Prognostic Models for Global Functional Outcome and Post-Concussion Symptoms Following Mild Traumatic Brain Injury: A Collaborative European NeuroTrauma Effectiveness Research in Traumatic Brain Injury (CENTER-TBI) Study
Source: J Neurotrauma. 2023 Aug 16;40(15-16):1651–70. doi: 10.1089/neu.2022.0320 (PMC10458380; doi:10.1089/neu.2022.0320)
Supplement: Supplemental data [file Supp_TableS3.docx]

**Supplementary Table 3. Selected models for Glasgow Outcome Scale Extended (GOSE) and persistent post-concussion symptoms (Rivermead Post-Concussion Symptoms Questionnaire, RPQ): model performance without correction for optimism**

|  | **GOSE 1-8, N=2376** | | | | | **GOSE 1-8, N=640** | | |
| --- | --- | --- | --- | --- | --- | --- | --- | --- |
|  | **Core** | **Clinical** | **Clinical+**  **early symptoms** | **Clinical+**  **CT** | **Clinical+**  **Biomarker** | **Clinical+**  **early symptoms, CT, biomarkers** | **Clinical+**  **2-3-wk symptoms** | **Clinical in a subset with 2-3- wk symptoms** |
| **C-statistic**  **95% CI** | 0.69[0.68-0.70] | 0.70[0.69-0.72] | 0.71[0.70-73] | 0.71[0.70-73] | 0.71[0.70-73] | 0.72[0.71-0.74] | 0.76[0.72-0.80] | 0.65[0.63-0.69] |
| **Nagelkerke R2** | 0.19 | 0.22 | 0.23 | 0.25 | 0.24 | 0.27 | 0.25 | 0.10 |
|  | **RPQ Total Score, N= 1605** | | | | | **RPQ Total Score, N=476** | | |
| **R2** | 0.05 | 0.12 | 0.14 | 0.12 | 0.12 | 0.15 | 0.42 | 0.11 |

**CT=Computed Tomography; R2=coefficient of determination (proportion of variance explained)**
