# Supplementary material for: Prognostic Models for Global Functional Outcome and Post-Concussion Symptoms Following Mild Traumatic Brain Injury: A Collaborative European NeuroTrauma Effectiveness Research in Traumatic Brain Injury (CENTER-TBI) Study
Source: J Neurotrauma. 2023 Aug 16;40(15-16):1651–70. doi: 10.1089/neu.2022.0320 (PMC10458380; doi:10.1089/neu.2022.0320)
Supplement: Supplemental data [file Supp_TableS4.docx]

**Supplementary Table 4. Odds ratios (95% confidence intervals) for predicting better 6-month functional outcome on Glasgow Outcome Scale Extended (GOSE)** **(N=2376): selected predictors**

| **Predictors of ordinal**  **GOSE (1-8)** | **Univariable analyses** | **Core model** | **Clinical model** | **Clinical +early symptoms (RPQ)** | **Clinical +CT** | **Clinical +Biomarkers** | **Clinical+ early RPQ, CT, biomarkers** | **Clinical+2-3wk symptoms**  **[subset N=640]** |
| --- | --- | --- | --- | --- | --- | --- | --- | --- |
| **Core variables (Odds ratios (OR))** | | | | | | | | |
| Age (25%:75%) | 0.49 [0.43-0.57] | 0.46[0.40-0.53] | 0.57[0.48-0.68] | 0.53[0.45-0.63] | 0.63[0.53-0.75] | 0.70[0.58-0.83] | 0.66[0.55-0.80] | 0.45[0.31-0.64] |
| ISS¹*(25%:75%) | 0.35 [0.31-0.40] | 0.38[0.33-0.43] | 0.36[0.32-0.41] | 0.37[0.32-0.42] | 0.43[0.37-0.49] | 0.45[0.39-0.52] | 0.48[0.41-0.56] | 0.41[0.26-0.64] |
| GCS 13:15 | 0.25 [0.19-0.34] | 0.43[0.31-0.58] | 0.42[0.31-0.57] | 0.43[0.31-0.58] | 0.56[0.41-0.77] | 0.49[0.36-0.67] | 0.59[0.43-0.81] | 0.69[0.26-1.81] |
| 14:15 | 0.51 [0.42-0.62] | 0.64[0.52-0.79] | 0.64[0.52-0.79] | 0.66[0.53-0.80] | 0.73[0.59-0.91] | 0.72[0.58-0.88] | 0.79[0.63-0.97] | 1.22[0.67-2.24] |
| **Clinical and sociodemographic variables** | | | | | | | | |
| Sex – Female: Male | 0.80 [0.69-0.93] | x | 0.72[0.61-0.85] | 0.76[0.65-0.90] | 0.71[0.60-0.84] | 0.74[0.63-0.88] | 0.77[0.65-0.92] | Not Selected |
| Psychiatric History | 0.55 [0.44-0.68] | x | 0.63[0.50-0.79] | 0.67[0.54-0.85] | 0.63[0.50-0.79] | 0.63[0.50-0.80] | 0.68[0.54-0.86] | 1.56[0.93-2.60] |
| ASAPS: no disease  mild | 0.56 [0.48-0.66] | x | 0.69[0.57-0.83] | 0.71[0.58-0.85] | 0.69[0.57-0.83] | 0.68[0.56-0.82] | 0.71[0.58-0.86] | Not Selected |
| severe | 0.32 [0.25-0.41] |  | 0.42[0.32-0.57] | 0.44[0.33-0.59] | 0.41[0.31-0.55] | 0.45[0.34-0.61] | 0.47[0.35-0.63] |  |
| Education: second. |  | x | Not Selected | x | x | x | x | x |
| none/primary | 0.72[0.55- 0.94] |  |  |  |  |  |  |  |
| program | 1.11[0.90- 1.38] |  |  |  |  |  |  |  |
| college/university | 1.26[1.03- 1.55] |  |  |  |  |  |  |  |
| Employment: full-t. |  | x | Not Selected | x | x | x | x | x |
| Part-time | 0.86[0.67-1.11] |  |  |  |  |  |  |  |
| Student | 1.62[1.21-2.16] |  |  |  |  |  |  |  |
| Retired | 0.56[0.46-0.67] |  |  |  |  |  |  |  |
| Unemployed | 0.62[0.47-0.81] |  |  |  |  |  |  |  |
| Living alone | 0.89 [0.75-1.07] | x | Not Selected | x | x | x | x | x |
| Prior TBI | 1.16[0.91- 1.48] | x | Not Selected | x | x | x | x | x |
| Preinjury migraines | 0.91[0.62- 1.34] | x | Not Selected | x | x | x | x | x |
| Cause: fall  traffic | 0.91[0.78-1.07] | x | 0.89[0.75-1.06] | 0.88[0.74-1.05] | 0.86[0.72-1.03] | 0.87[0.73-1.03] | 0.80[0.67-0.96] | 0.56[0.38-0.81] |
| violence | 0.96[0.68- 1.34] |  | 0.62[0.44-0.88] | 0.66[0.46-0.93] | 0.62[0.43-0.88] | 0.63[0.44-0.91] | 0.70[0.48-1.00] | 1.30[0.49-3.44] |
| Alcohol intoxication | 0.99 [0.82-1.19] | x | Not Selected | x | x | x | x | x |
| Pupils | 0.42[0.26- 0.67] | x | 0.52[0.33-0.84] | 0.54[0.34-0.86] | 0.52[0.32-0.82] | 0.51[0.32-0.83] | 0.54[0.34-0.87] | Not Selected |
| PTA: no  <2h | 1.20[0.99- 1.46] | x | Not Selected | x | x | x | x | x |
| >2 | 0.64[0.51- 0.79] |  |  |  |  |  |  |  |
| LOC | 1.11[0.95-1.30] | x | Not Selected | x | x | x | x | x |
| Vomiting | 0.82 [0.67-1.00] | x | Not Selected | x | x | x | x | x |
| Headache | 1.35[1.13- 1.62] | x | Not Selected | x | x | x | x | x |
| **Early RPQ *** | 0.74[0.67- 0.82] | x | x | 0.74[0.67-0.82] | x | x | 0.74[0.66-0.82] | x |
| **CT variables** | | | | | | | | |
| Any abnormality | 0.33[0.28-0.39] | x | x | x | 0.74[0.58-0.95] | x | 0.79[0.60-1.03] | x |
| TAI | 0.70[0.48- 1.00] | x | x | x | 1.38[0.93-2.05] | x | 1.19[0.93-1.52] | x |
| Midline shift | 0.28 [0.18-0.42] | x | x | x | Not Selected | x | x | x |
| Cistern compress. | 0.23 [0.16-0.33] | x | x | x | Not Selected | x | x | x |
| tSAH | 0.33 [0.28-0.40] | x | x | x | 0.73[0.57-0.94] | x | 0.75[0.58-0.98] | x |
| Contusion | 0.42 [0.35-0.51] | x | x | x | Not Selected | x | Not Selected | x |
| Nonevacuated hem. | 0.22 [0.16-0.29] | x | x | x | 0.48[0.34-0.66] | x | 0.52[0.38-0.73] | x |
| **Biomarkers** | | | | | | | | |
| log GFAP* | 0.43[0.37-0.49] | x | x | x | x | Not Selected | 1.19[0.94-1.50] | x |
| log NSE* | 0.83[0.77-0.91] | x | x | x | x | 1.09[0.99-1.20] | 1.09[0.99-1.22] | x |
| log NFL* | 0.42[0.38-0.47] | x | x | x | x | 0.71[0.62-0.81] | 0.75[0.63-0.88] | x |
| log S100B* | 0.53[0.47-0.59] | x | x | x | x | 0.76[0.67-0.87] | 0.75[0.65-0.87] | x |
| log Total-Tau* | 0.50[0.45-0.56] | x | x | x | x | Not Selected | Not Selected | x |
| log UCHL1* | 0.49[0.43-0.55] | x | x | x | x | Not Selected | Not Selected | x |
| **Symptoms at 2-3 weeks** | | | | | | | | |
| RPQ* | 0.31[0.25-0.40] | x | x | x | x | x | x | 0.37[0.26-0.52] |
| PCL-5* | 0.49[0.41-0.59] | x | x | x | x | x | x | 0.71[0.55-0.91] |
| PHQ-9* | 0.40[0.31- 0.52] | x | x | x | x | x | x | Not Selected |
| GAD-7* | 0.52[0.42-0.64] | x | x | x | x | x | x | Not Selected |

*Legend: ¹nonlinear; *OR scaled to correspond to 25:75 percentile of continuous predictors; ** only 10 outcome events; C= concordance index; GCS= Glasgow Coma Score; ISS= Injury Severity Score Total; LOC= Loss of consciousness; RPQ= Rivermead Post-Concussion Symptoms Questionnaire; PCL-5= Post-Traumatic Stress Disorder (PTSD) Checklist for DSM-5; GAD-7= Generalized Anxiety Disorder 7-item scale (GAD-7); PHQ-9= Patient Health Questionnaire; PTA= Posttraumatic amnesia; TAI= Traumatic axonal injury; tSah= Traumatic subarachnoid hemorrhage.*
