# Supplementary material for: Prognostic Models for Global Functional Outcome and Post-Concussion Symptoms Following Mild Traumatic Brain Injury: A Collaborative European NeuroTrauma Effectiveness Research in Traumatic Brain Injury (CENTER-TBI) Study
Source: J Neurotrauma. 2023 Aug 16;40(15-16):1651–70. doi: 10.1089/neu.2022.0320 (PMC10458380; doi:10.1089/neu.2022.0320)
Supplement: Supplemental data [file Supp_FigS1.docx]

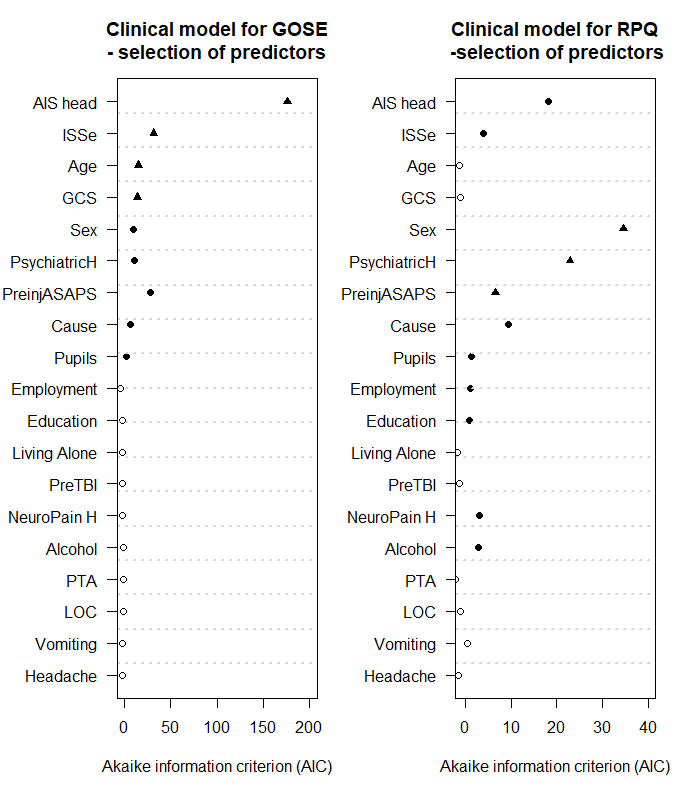


**Suppl. Figure 1.** Clinical model for Glasgow Outcome Scale Extended (GOSE) and Rivermead Post-Concussion Symptoms Questionnaire (RPQ): Abbreviated Injury Score (AIS) for head and Injury Severity Score (ISS) for extra-cranial injury instead of Total ISS

*Black circles indicate selected predictors based on AIC. Black triangles indicate pre-specified core predictors.*

*Legend:* AIS= Abbreviated Injury Score; ASA-PS= American Society of Anesthesiologists Physical Status; GCS= Glasgow Coma Scale; ISSe=Injury Severity Score Extra-cranial; Neuropain H=History of Migraines/ Headaches; PreTBI= Prior traumatic brain injury; PTA= Posttraumatic amnesia; LOC= Loss of consciousness.
