# Supplementary material for: Prognostic Models for Global Functional Outcome and Post-Concussion Symptoms Following Mild Traumatic Brain Injury: A Collaborative European NeuroTrauma Effectiveness Research in Traumatic Brain Injury (CENTER-TBI) Study
Source: J Neurotrauma. 2023 Aug 16;40(15-16):1651–70. doi: 10.1089/neu.2022.0320 (PMC10458380; doi:10.1089/neu.2022.0320)
Supplement: Supplemental data [file Supp_FigS5.docx]

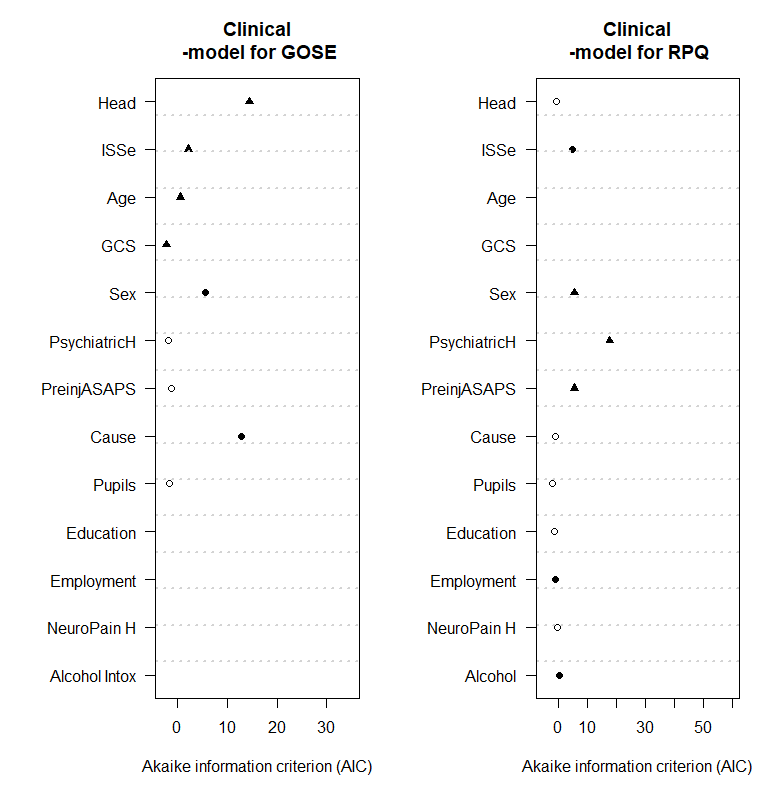
 **A)**

**
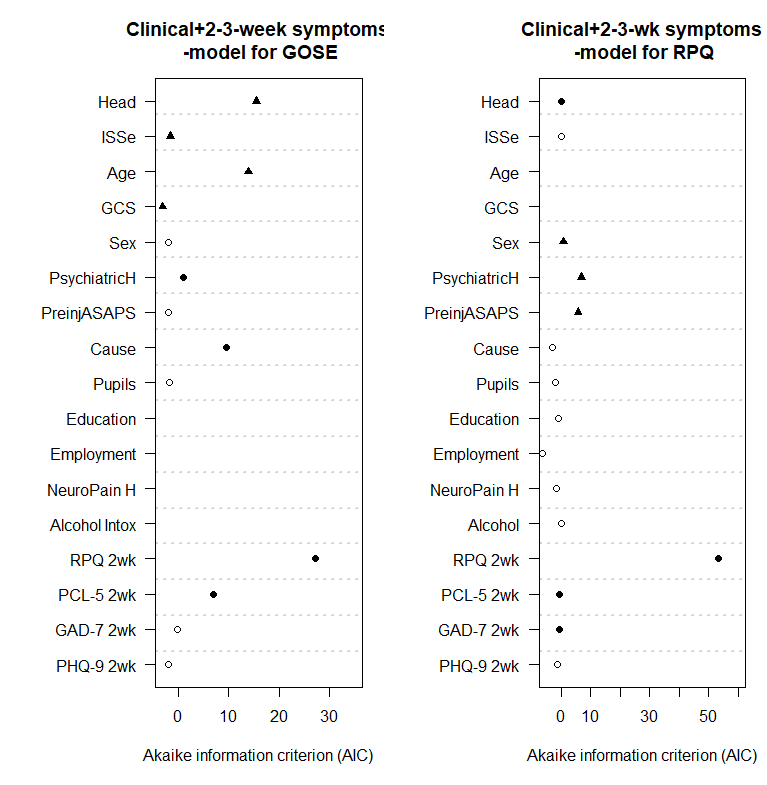
 B)**

Supp. Figure 5. The Clinical models (A) and Clinical+ 2-3 week symptoms models (B) for prediction of Glasgow Outcome Scale Extended (GOSE) (N=640) and Rivermead Postconcussion Symptom Questionnaire (RPQ) (N=476) : Abbreviated Injury Score (AIS) for head and Injury Severity Score (ISS) for extra-cranial injury instead of Total ISS

*Black circles indicate selected predictors based on AIC. Black triangles indicate pre-specified core predictors.*

Legend: ASA-PS= American Society of Anesthesiologists Physical Status; GCS= Glasgow Coma Scale; Neuropain H=History of Migraines/ Headaches; RPQ= Rivermead Post-Concussion Symptoms Questionnaire; PCL-5= Post-Traumatic Stress Disorder (PTSD) Checklist for DSM-5; GAD-7= Generalized Anxiety Disorder 7-item scale (GAD-7); PHQ-9= Patient Health Questionnaire.
