# Supplementary material for: Prognostic Models for Global Functional Outcome and Post-Concussion Symptoms Following Mild Traumatic Brain Injury: A Collaborative European NeuroTrauma Effectiveness Research in Traumatic Brain Injury (CENTER-TBI) Study
Source: J Neurotrauma. 2023 Aug 16;40(15-16):1651–70. doi: 10.1089/neu.2022.0320 (PMC10458380; doi:10.1089/neu.2022.0320)
Supplement: Supplemental data [file Supp_FigS6.docx]

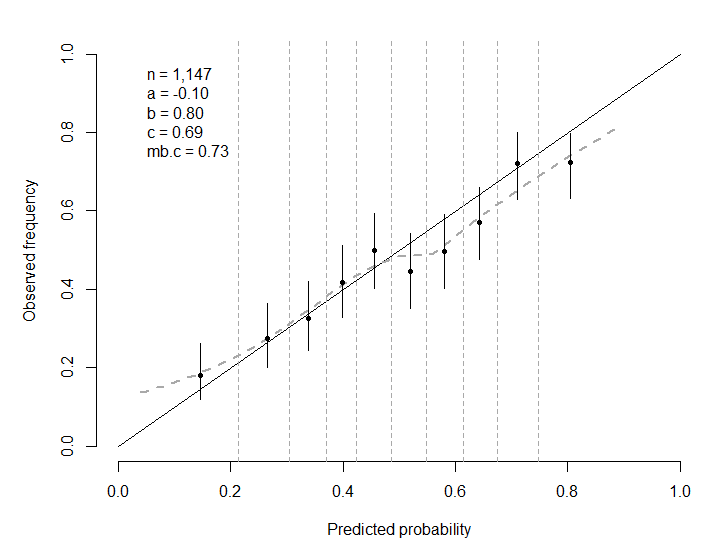
 A)
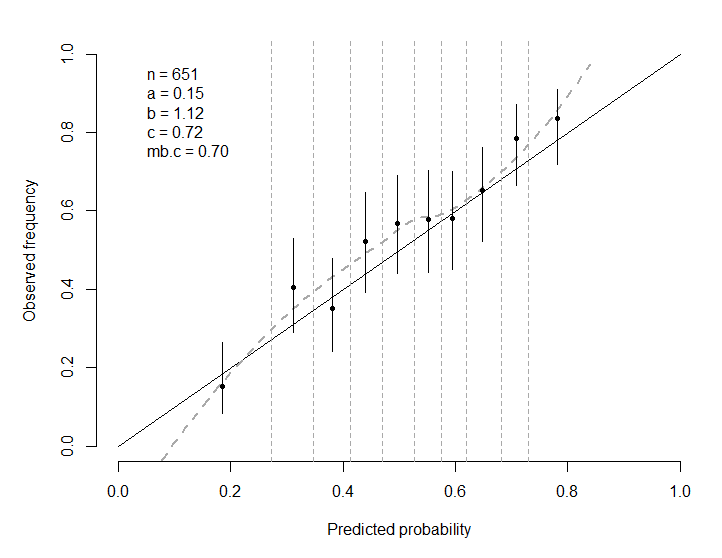
B)


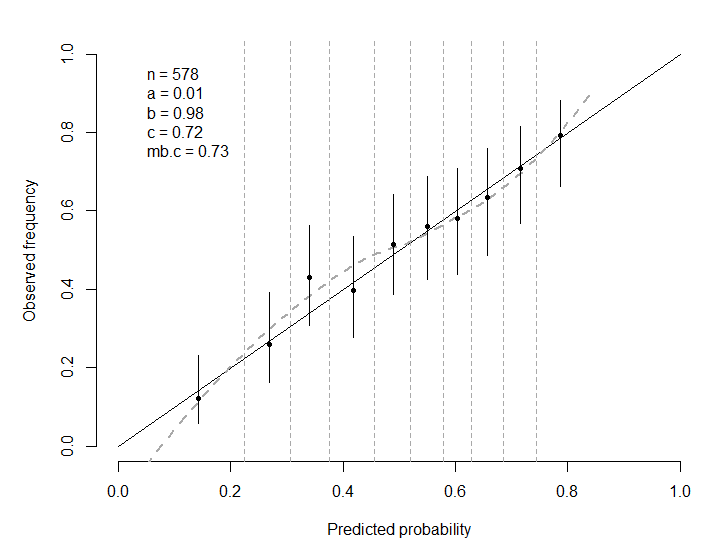
 C)

Suppl. Figure 6. Calibration plots for Clinical model predicting GOSE==8 in regions West (A), North (B), South-East (C): cross-validation with leave one region out method. West: AT, NL, BE, DE, GB, FR. North: FI, NO, SE, DK, LV, LT. South-East: IT, ES, IL, HU, RO, SR.
