# Supplementary material for: Prognostic Models for Global Functional Outcome and Post-Concussion Symptoms Following Mild Traumatic Brain Injury: A Collaborative European NeuroTrauma Effectiveness Research in Traumatic Brain Injury (CENTER-TBI) Study
Source: J Neurotrauma. 2023 Aug 16;40(15-16):1651–70. doi: 10.1089/neu.2022.0320 (PMC10458380; doi:10.1089/neu.2022.0320)
Supplement: Supplemental data [file Supp_TableS5.docx]

**Supplementary Table 5. Regression coefficients (95% confidence intervals) for predicting 6-month persistent post-concussion symptoms (Rivermead Post-concussion Symptoms Questionnaire (RPQ) total score) in mild TBI (N=1605): selected predictors**

| **Predictors of RPQ at 6 months** | **Univariable analyses** | **Core model** | **Clinical model** | **Clinical +early symptoms (RPQ)** | **Clinical +CT** | | **Clinical +Biomarker** | **Clinical+ early symptoms ,CT, biomarkers** | | **Clinical+2-3wk symptoms**  **[subset N=476]** |
| --- | --- | --- | --- | --- | --- | --- | --- | --- | --- | --- |
| **Clinical and sociodemographic variables** | | | | | | | | | | |
| Age¹ | -0.18[-1.29,0.93] | x | Not Selected | x | x | | x | x | | x |
| GCS 13:15 | 4.27 [1.76, 6.77] | x | Not Selected | x | x | | x | x | | x |
| 14:15 | 2.49[0.85, 4.13] | x |  |  |  | |  |  | |  |
| ISS ¹ | 2.80[1.87,3.74] | x | 2.77[1.85,3.70] | 2.56[1.64,3.47] | 2.31[1.31,3.31] | | 3.07[1.97,4.16] | 2.59[1.50,3.68] | | 1.74[-0.58,4.07] |
| Education: second.  none/primary | -0.62 [-2.30,1.46] | x | -0.31[-2.29,1.67] | -0.22[-2.19,1.75] | -0.40[-2.38,1.57] | | -0.26[-2.23,1.71] | -0.37[-2.33,1.59] | | Not Selected |
| program | -0.57 [-2.30, 1.17] |  | 0.54[-1.07,2.16] | 0.32[-1.28,1.92] | 0.56[-1.05,2.17] | | 0.55[-1.07,2.17] | 0.31[-1.29,1.91] | |  |
| college/university | -2.15[-3.79, -0.51] |  | -1.72[-3.26,-0.18] | -2.00[-3.54,-0.46] | -1.72[-3.26,-0.18] | | -1.69[-3.25,-0.14] | -2.05[-3.60,-0.51] | |  |
| Employment: full-t. |  | x |  |  | |  |  | |  | Not Selected |
| Part-time |  |  | 0.22[-1.71,2.14] | 0.14[-1.75,2.02] | | 0.18[-1.75,2.10] | 0.31[-1.62,2.25] | | 0.21[-1.68,2.10] |  |
| Student |  |  | -1.79[-3.99,0.42] | -2.16[-4.37,0.05] | | -1.81[-4.01,0.39] | -1.79[-3.99.0.41] | | -2.11[-4.31,0.08] |  |
| Retired |  |  | -2.16[-3.82,-0.51] | -1.60[-3.24,0.04] | | -2.32[-3.97,-0.66] | -2.03[-3.69,-0.37] | | -1.62[-3.27,0.04] |  |
| Unemployed |  |  | 3.04 [0.58,5.50] | 2.72[0.30,5.14] | | 2.97[0.51,5.43] | 3.00 [0.54,5.46] | | 2.71[0.30,5.13] |  |
| Living alone | 0.3 [-1.2, 1.79] | x | Not Selected | x | x | | x | x | | Not Selected |
| Prior TBI | 0.48 [-1.48, 2.44] | x | Not Selected | x | x | | x | x | | Not Selected |
| Preinjury migraines | 6.18[3.1, 9.26] | x | 3.75[0.76,6.75] | 2.87[-0.10,5.84] | 3.79[0.80,6.78] | | 3.65[0.65,6.65] | 2.90[-0.07,5.86] | | Not Selected |
| Cause: fall  traffic | 2.32[1.04, 3.6] | x | 2.25[0.96,3.53] | 2.35[1.08,3.62] | 2.34[1.06,3.63] | | 2.40[1.11,3.70] | 2.49[1.22,3.76] | | Not Selected |
| violence | 3.43[0.55, 6.32] |  | 2.10[-0.75,4.94] | 1.28[-1.54,4.10] | 2.14[-0.69,4.98] | | 1.85[-1.01,4.70] | 1.09[-1.74,3.91] | | Not Selected |
| Alcohol intoxication | 2.62[1.08, 4.15] | x | 2.10[0.54,3.65] | 2.03[0.49,3.57] | 2.05[0.50,3.61] | | 2.12[0.56,3.67] | 2.01[0.48,3.54] | | Not Selected |
| Pupils | 5.66[ 1.13, 10.19] | x | 4.42[0.02,8.81] | 3.91[-0.61,8.44] | 4.38[-0.03,8.79] | | 4.17[-0.23,8.57] | 3.77[-0.74,8.29] | | Not Selected |
| PTA: no  <2h | -0.65[-2.09, 0.80] | x | Not Selected | x | x | | x | x | | x |
| >2 | 0.79[-1.04, 2.61] |  |  |  |  | |  | x | |  |
| LOC | -0.82 [-2.10, 0.46] | x | Not Selected | x | x | | x | x | | x |
| Vomiting | -0.49 [-2.15, 1.17] | x | Not Selected | x | x | | x | x | | x |
| Headache | 0.21 [-1.26, 1.69] | x | Not Selected | x | x | | x | x | | x |
| **Early RPQ *** | 3.4 [2.6, 4.21] | x | x | 2.64[1.83,3.45] | x | | x | 2.59[1.78,3.40] | | x |
| **CT variables** | | | | | | | | | | |
| Any abnormality | 2.61[1.37, 3.84] | x | x | x | 1.54[0.23,2.84] | | x | 1.90[0.39,3.42] | | x |
| TAI |  | x | x | x | Not Selected | | x | Not Selected | | x |
| Midline shift | 2.56 [-0.94, 6.1] | x | x | x | Not Selected | | x | Not Selected | | x |
| Cistern. Compress. | 1.94 [-1.26, 5.2 ] | x | x | x | Not Selected | | x | Not Selected | | x |
| tSAH | 2.49 [1.17, 3.81] | x | x | x | Not Selected | | x | Not Selected | | x |
| Contusion | 1.99 [0.46, 3.53] | x | x | x | Not Selected | | x | Not Selected | | x |
| Nonevacuated hem. | 2.28 [-0.25, 4.81] | x | x | x | Not Selected | | x | Not Selected | | x |
| **Biomarkers** | | | | | | | | | | |
| log GFAP | 0.88 [-0.19, 1.96] | x | x | x | x | | 0.22[-1.25,1.70] | -0.79[-2.46,0.88] | | x |
| log NSE | 0.47[-0.20, 1.14] | x | x | x | x | | x | Not Selected | | x |
| log NFL | 1.04[0.14, 1.93] | x | x | x | x | | x | Not Selected | | x |
| log S100B | 1.05 [0.14, 1.95] | x | x | x | x | | x | Not Selected | | x |
| log Total-Tau | 0.63 [-0.19, 1.45] | x | x | x | x | | -0.87[-1.99,0.26] | Not Selected | | x |
| log UCHL1 | 0.56 [-0.28,1.40] | x | x | x | x | | x | Not Selected | | x |
| **2-week symptoms** | | | | | | | | | | |
| RPQ | 11.12[9.7, 12.52] | x | x | x | x | | x | x | | 7.82[5.98,9.66] |
| PCL-5 | 7.42[6.29, 8.57] | x | x | x | x | | x | x | | 1.79[-0.04,3.61] |
| PHQ-9 | 9.75[8.28, 11.22] | x | x | x | x | | x | x | | Not Selected |
| GAD-7 | 7.2[6.07, 8.40] | x | x | x | x | | x | x | | 1.85[-0.34,4.03] |

*Legend: ¹nonlinear terms with polynomials; C= concordance index; R2= coefficient of determination; GCS= Glasgow Coma Score; ISS= Injury Severity Score Total; LOC= Loss of consciousness; RPQ= Rivermead Post-Concussion Symptoms Questionnaire; PCL-5= Post-Traumatic Stress Disorder (PTSD) Checklist for DSM-5; GAD-7= Generalized Anxiety Disorder 7-item scale (GAD-7); PHQ-9= Patient Health Questionnaire; PTA= Posttraumatic amnesia; TAI= Traumatic axonal injury; tSah= Traumatic subarachnoid hemorrhage.*
