# Supplementary material for: Prognostic Models for Global Functional Outcome and Post-Concussion Symptoms Following Mild Traumatic Brain Injury: A Collaborative European NeuroTrauma Effectiveness Research in Traumatic Brain Injury (CENTER-TBI) Study
Source: J Neurotrauma. 2023 Aug 16;40(15-16):1651–70. doi: 10.1089/neu.2022.0320 (PMC10458380; doi:10.1089/neu.2022.0320)
Supplement: Supplemental data [file Supp_TableS6.docx]

**Supplementary Table 6. Equations for models for Glasgow Outcome Scale Extended (1-8)**

1. **Ordinal outcome**

The probability of GOSE at a particular threshold can be predicted by **1/(1 + exp ^– Model^)**, with Model defined according to one of the models listed below: :

**Core**= Intercept¹+ (-0.022 *Age)+( 0.408 *1 ^GCS=14^) + (0.844*1 ^GCS=15^) + (0.127* log(ISS))+ (-0.195*log(ISS)^2)

¹4.923 GOSE >=3; 4.066 GOSE>=4; 3.656 GOSE>=5; 2.977 GOSE>=6; 2.303 GOSE >=7; 1.238 GOSE >=8

**Clinical**=Intercept¹+(-0.015*Age)+(0.407*1^GCS=14^)+(0.829*1^GCS=15^)+(0.038*log(ISS))+(-0.175*log(ISS)^2)+(0.315*1^Sex=Male^)+(-0.439*1^PsychiatriHistory^)+(-0.354*1^ASAPS=Mild systemic disease^)+(-0.817*1^ASAPS =Severe systemic disease^)+(-0.110*^Cause= traffic^)+(-0.452*1^Cause= violence^)+(-0.616*1 ^Pupil(s)=Nonreactive^)

¹4.904 GOSE >=3; 4.029 GOSE>=4; 3.608 GOSE>=5; 2.908 GOSE>=6; 2.213 GOSE >=7; 1.127 GOSE >=8

**Clinical+ early symptoms=**Intercept¹+(-0.018*Age)+(0.422*1^GCS=14^)+(0.835*1^GCS=15^)+(0.043*log(ISS))+(-0.179*log(ISS)^2)+(0.262*1^Sex=Male^)+(-0.386*1 ^PsychiatriHistory=Yes^)+(-0.341*1^ASAPS=mild systemic disease^)+(-0.799*1^ASAPS=severe systemic disease^)+(-0.125*1^Cause= traffic^)+(-0.413*1^Cause=violence^)+( -0.604 *1 ^Pupil(s)=Nonreactive^)+(0.027* RPQ Total Score Early)

¹5.374 GOSE >=3; 4.496 GOSE>=4; 4.072 GOSE>=5; 3.368 GOSE>=6; 2.665 GOSE >=7; 1.559 GOSE >=8

**Clinical+ CT**=Intercept¹+(-0.013*Age)+(0.262*1^GCS=14^)+(0.561*1^GCS=15^)+(0.135*log(ISS))+(-(0.172*log(ISS) ^2)+(0.334*1 ^Sex=Male^)+(- 0.442*1 ^PsychiatriHistory^)+(-0.360*1 ^ASAPS=Mild systemic disease^ 0.860*1 ^ASAPS =Severe systemic disease^)+( -0.625 *1 ^Pupil(s)=Nonreactive^)+(-0.193 *1 ^Cause= traffic^)+**(**-0.416* ^Cause= violence^)+(-0.290*1 ^Any Intracranial Traumatic Abnormality^) + (0.312* 1^Traumatic Axonal Injury^)+(-0.718*1^Non-evacuated Hematoma^)+ **(**-0.302 *1^Traumatic Subarachnoid Hemorrhage^)

¹5.178 GOSE >=3; 4.274 GOSE>=4; 3.839 GOSE>=5; 3.119 GOSE>=6; 2.407 GOSE >=7; 1.292 GOSE >=8

**Clinical+ biomarkers=**Intercept¹+(-0.010*Age)+(0.369*1^GCS=14^)+(0.693*1^GCS=15^)+(-0.051*log(ISS))+(-0.122* log(ISS) ^2)+(0.288*1 ^Sex=Male^)+(-0.442*1 ^PsychiatriHistory^)+(-0.374*1 ^ASAPS=Mild systemic disease^)+( -0.765*1 ^ASAPS =Severe systemic disease^)+( -0.651*1 ^Pupil(s)=Nonreactive^)+( -0.146* 1 ^Cause= traffic^)+( -0.469*1 ^Cause= violence^)+(-0.226* log S100B)+( -0.236 *log NFL)+( 0.147*log NSE)

¹ 4.494 GOSE >=3; 3.599 GOSE>=4; 3.166 GOSE>=5; 2.448 GOSE>=6; 1.739 GOSE >=7; 0.632 GOSE >=8

**Clinical+ early symptoms, CT, biomarker=** Intercept¹ +(-0.011 *Age) + (0.276 * ^GCS=14^)+ (0.506* ^GCS=15^)+ (0.011* log(ISS))+( -0.124* log(ISS) ^2) + (0.243* 1 ^Sex=Male^)+(- 0.370 *1 ^PsychiatriHistory^) +(-0.329 *1 ^ASAPS=Mild systemic disease^)+( -0.727* 1 ^ASAPS =Severe systemic disease^)+( -0.588 * 1 ^Pupil(s)=Nonreactive^)+( -0.209 *1 ^Cause= traffic^) + (-0.345* 1 ^Cause= violence^)+( -0.230 *1 ^Any Intracranial Traumatic Abnormality^)+( -0.615* 1^Non-evacuated Hematoma^)+(-0.269 *1^Traumatic Subarachnoid Hemorrhage^)+( 0.154+log NSE)+(-0.199* log NFL)+( -0.235* log S100B) +(0.059 *log GFAP )+(-0.027 *RPQ Total Score Early)

¹ 5.017 GOSE >=3; 4.104 GOSE>=4; 3.663 GOSE>=5; 2.936 GOSE>=6; 2.214 GOSE >=7; 1.079 GOSE >=8

**Clinical+ 2-3 week symptoms=** Intercept¹ +**(**-0.020*Age)+(0.511*1 ^GCS=14^)+ (0.330*1 ^GCS=15^) +(0.268* log(ISS))+(-0.198* log(ISS) ^2)+(0.394 *1 ^PsychiatriHistory^)+ ( -0.525*1 ^Cause= traffic^) +(0.232*1 ^Cause= violence^)+( -0.044 *RPQ Total Score 2-3wks)+( -0.018* PCL5 Total Score 2-3wks)

¹ 8.777 GOSE >=3; 6.542 GOSE>=4; 6.432 GOSE>=5; 5.024 GOSE>=6; 3.923 GOSE >=7; 2.493 GOSE >=8

1. **Outcome Complete return to preinjury functioning (GOSE=8)**

**Core**= 1.076+(-0.014 *Age)+( 0.290*1 ^GCS=14^) + (0.585* 1^GCS=15^) + (0.149* log(ISS))+ (-0.200 *log(ISS)^2)

**Clinical**=0.965+(-0.010*Age)+(0.269*1^GCS=14^)+(0.549*1^GCS=15^)+(0.099*log(ISS))+(-0.193*log(ISS)^2)+(0.403*1^Sex=Male^)+(-0.533*1^PsychiatricHistory=Yes^)+(-0.263*1^ASAPS=Mild systemic disease^)+(-0.451*1^ASAPS=Severe systemic disease^)+(-0.169*1^Cause=traffic^)+(-0.190*1^Cause=violence^)+(-0.502*1^Pupil(s)=Nonreactive^)

**Clinical+ early symptoms=** 1.569+ (-0.013 *Age)+(0.280*1 ^GCS=14^) +(0.523*1 ^GCS=15^)+(0.090* log(ISS))+(-0.194* log(ISS) ^2) +( 0.340* 1 ^Sex=Male^)+(-0.463*1 ^PsychiatriHistory=Yes^)+( -0.243* 1 ^ASAPS=Mild systemic disease^)+(-0.422*1 ^ASAPS =Severe systemic disease^)+(-0.192*1 ^Cause= traffic^)+(-0.139* 1 ^Cause= violence^)+(-0.495 * 1 ^Pupil(s)=Nonreactive^)+(-0.037* RPQ Total Score Early)

**Clinical+ CT**=1.127+(-0.007*Age)+(0.114*1 ^GCS=14^)+(0.310*1 ^GCS=15^)+(0.174*log(ISS))+(-(0.181*log(ISS) ^2)+(0.416*1 ^Sex=Male^)+(- 0.537*1 ^PsychiatriHistory=Yes^)+(-0.263*1 ^ASAPS=Mild systemic disease^)+(-0.496* 1 ^ASAPS =Severe systemic disease^)+(-0.534*1 ^Pupil(s)=Nonreactive^)+(-0.241*1=Cause traffic)+**(**-0. 163*1 ^Cause= violence^)+(-0.338*1 ^Any Intracranial Traumatic Abnormality^) + (0.336* 1^TAI^)+(-0.379*1^Non-evacuated Hematoma^)+ **(**-0.340 *1^Traumatic Subarachnoid Hemorrhage^)

**Clinical+ biomarkers=** 0.533+(-0.005*Age)+(0.218*1 ^GCS=14^)+(0.424*1 ^GCS=15^)+(0.022*log(ISS))+(-(0.148*log(ISS) ^2)+(0.384*1 ^Sex=Male^)+(- 0.543*1 ^PsychiatriHistory=Yes^)+(-0.273*1 ^ASAPS=Mild systemic disease^)+(-0.407* 1 ^ASAPS =Severe systemic disease^)+(-0.522*1 Nonreactive pupil(s))+(-0.189*1 ^Cause= traffic^)+**(**-0.226*1 ^Cause= violence^) +(-0.211* log S100B)+( -0.180 *log NFL)+( 0.097*log NSE)

**Clinical+ early symptoms, CT, biomarker=** 1.078 +(-0.007 *Age) + (0.115 * 1^GCS=14^)+ (0.230* ^GCS=15^)+ (0.061* log(ISS))+( -0.140* log(ISS) ^2) + (0.312* 1 ^Sex=Male^)+(- 0.445 *1 ^PsychiatriHistory=Yes^) +(-0.222 *1 ^ASAPS=Mild systemic disease^)+( -0.383* 1 ^ASAPS =Severe systemic disease^)+( -0.486 * 1 ^Pupil(s)=Nonreactive^)+( -0.251 *1 ^Cause= traffic^) + (-0.113* ^Cause= violence^)+( -0.268 *1 ^Any Intracranial Traumatic Abnormality^)+(-0.297* 1 ^Non-evacuated Hematoma^)+(-0.316 *1 ^Traumatic Subarachnoid Hemorrhage^)+ (0.123*1 log NSE)+(-0.138* log NFL)+( -0.227* logS100B) +(0.044*log GFAP )+(-0.036 *RPQ Total Score Early)

**Clinical+ 2-3 week symptoms=** 2.912 +**(**-0.020*Age)+(0.308*1 ^GCS=14^)+ (-0.045*1 ^GCS=15^) +(0.325* log(ISS))+(-0.232* log(ISS) ^2)+(0.351 *1 ^PsychiatriHistory=Yes^)+ ( -0.541*1 ^Cause= traffic^) +(0.483*1 ^Cause= violence^)+( -0.050 *RPQ Total Score 2-3wks)+( -0.019* PCL5 Total Score 2-3wks)
