# Supplementary material for: Prognostic Models for Global Functional Outcome and Post-Concussion Symptoms Following Mild Traumatic Brain Injury: A Collaborative European NeuroTrauma Effectiveness Research in Traumatic Brain Injury (CENTER-TBI) Study
Source: J Neurotrauma. 2023 Aug 16;40(15-16):1651–70. doi: 10.1089/neu.2022.0320 (PMC10458380; doi:10.1089/neu.2022.0320)
Supplement: Supplemental data [file Supp_TableS7.docx]

**Supplementary Table 7. Equations for models for persistent post-concussion symptoms (RPQ Total Score)**

**Core**= 11.031 +(-2.825*1 ^Sex=Male^)+(6.073*1 ^PsychiatriHistory=Yes^)+(0.617 *1 ^ASAPS=Mild systemic disease^) + (2.296*1 ^ASAPS =Severe systemic disease^)

**Clinical**=7.295+(-3.376*1 ^Sex=Male^)+ (4.460*1^PsychiatriHistory=Yes^)+(1.098*1^ASAPS=Mild systemic disease^)+(3.138*1 ^ASAPS =Severe systemic disease^)+(-0.709*log(ISS))+(0.584*log(ISS)^2)+ (3.910*1 ^Pupil(s)=Nonreactive^)+ 1.988*1 ^Cause= traffic^)+(1.857*1 ^Cause= violence^) + (1.249*1 ^Education=None/primary school^)+(2.005*1 ^Education=In program^)+(1.524*1 ^Education=Secondary school^)+ (0.192*1 ^Employment=Part-time^)+(-1.916*1 ^Employment=Retired^)+(-1.584*1 ^Employment=Student^) +(2.690*1 ^Employment=Unemployed^)+ (3.323*1^History of headaches^) + (1.855*1^Alcohol intoxication^)

**Clinical+ early symptoms=**4.666+(-3.074*1^Sex=Male^)+ (4.384*1^PsychiatriHistory=Yes^)+(1.032*1 ^ASAPS=Mild systemic disease^)+(3.049*1 ^ASAPS =Severe systemic disease^)+ (-0.662*log(ISS))+(0.560*log(ISS)^2)+(3.641*1 ^Pupil(s)=Nonreactive^)+(2.187*1 ^Cause= traffic^)+ (1.189*1 ^Cause= violence^ =Cause violence) + (1.658*1 ^Education=None/primary school^)+ (2.161*1 ^Education=In program^)+(1.862*1 ^Education=Secondary school^)+ (0.128*1 ^Employment=Part-time^)+ (-1.487*1^Employment=Retired^)+( -2.010 *1^Employment=Student^) +(2.531*1^Employment=Unemployed^) +( 2.667*1 ^History of headaches^) + (1.887 *1 ^Alcohol intoxication^) +(0.223* RPQ Total Score Early)

**Clinical+ CT=**7.237+(-3.415*1 ^Sex=Male^)+ (4.480*1 ^PsychiatriHistory=Yes^)+(1.096* 1 ^ASAPS=Mild systemic disease^)+(3.265*1 ^ASAPS =Severe systemic disease^)+(-0.920*log(ISS))+(0.564*log(ISS)^2)+ (3.925*1^Pupil(s)=Nonreactive^)+ 2.100*1^Cause= traffic^)+ (1.922*1 ^Cause= violence^) + (1.180*1 ^Education=None/primary school^)+ (2.043*1 ^Education=In program^)+(1.542*1 ^Education=Secondary school^)+ (0.158*1 ^Employment=Part-time^)+( -2.08*1^Employment=Retired^)+(-1.624*1^Employment=Student^)+(2.665*1 ^Employment=Unemployed^)+(3.400*1^History of headaches^)+(1.840*1^Alcohol intoxication^)+(1.379*1^Any Intracranial Traumatic Abnormality^)

**Clinical+ biomarkers=**6.645+(-3.428*1^Sex=Male^)+(4.436*1^PsychiatriHistory=Yes^)+(1.135*1^ASAPS=Mild systemic disease^)+(3.249*1^ASAPS=Severe systemic disease^)+(-0.670*log(ISS))+(0.627*log(ISS)^2)+(3.746*1^Pupil(s)=Nonreactive^)+(2.160*1^Cause=traffic^)+ (1.660*1^Cause=violence^)+(1.289*1^Education=None/primary school^)+(2.016*1 ^Education=In program^)+(1.523 *1 ^Education=Secondary school^)+ (0.280* 1 ^Employment=Part-time^)+ (-1.828* 1 ^Employment=Retired^)+( -1.609*1 ^Employment=Student^) +(2.699*1 ^Employment=Unemployed^) +( 3.282*1 ^History of headaches^) + (1.901* 1 ^Alcohol intoxication^)+(-0.028*GFAP)+(0.171*GFAP^2)+(-0.581*Tau)

**Clinical+ all=**4.469+(-2.988*^Sex=Male^)+(4.120*1^PsychiatriHistory=Yes^)+(0.940*1^ASAPS=Mild systemic disease^)+(2.933*1^ASAPS=Severe systemic disease^)+(–0.766*log(ISS))+(0.556*log(ISS)^2)+(3.417*1^Pupil(s)=Nonreactive^)+(2.207*1 ^Cause= traffic^)+(0.950*1 ^Cause= violence^) +( 1.469* 1 ^Education=None/primary school^)+ (2.086*1 ^Education=In program^)+(1.794= 1 ^Education=Secondary school^)+ (0.153*1 ^Employment=Part-time^)+(-1.462*1 ^Employment=Retired^)+( -1.897 *1 ^Employment=Student^)+2.384*(1 ^Employment=Unemployed^) + (1.805*1 ^Alcohol intoxication^) + (2.637 * 1 ^History of headaches^) +(0.210* RPQ Total Score Early)+(1.704*1^Any Intracranial Abnormality^)+( - 0.331* log(GFAP))

**Clinical+ 2-3 weeks symptoms =** -2.396 +(1.795* ^Sex=Male^)+( 4.045* 1 ^PsychiatriHistory=Yes^)+(1.596* 1 ^ASAPS=Mild systemic disease^)+(5.155* 1 ^ASAPS =Severe systemic disease^) +(1.053*log(ISS))+ (0.051*log(ISS)^2)+(0.099*PCL5 Total Score 2-3wks)+( 0.369*RPQ Total Score 2-3wks)+(0.249*GAD7 Total Score 2-3wks)
